# Interaction between Galectin-3 and Integrins Mediates Cell-Matrix Adhesion in Endothelial Cells and Mesenchymal Stem Cells

**DOI:** 10.3390/ijms22105144

**Published:** 2021-05-13

**Authors:** Antonín Sedlář, Martina Trávníčková, Pavla Bojarová, Miluše Vlachová, Kristýna Slámová, Vladimír Křen, Lucie Bačáková

**Affiliations:** 1 Laboratory of Biomaterials and Tissue Engineering, Institute of Physiology of the Czech Academy of Sciences, Vídeňská 1083, CZ 142 20 Prague 4, Czech Republic; antonin.sedlar@fgu.cas.cz (A.S.); martina.travnickova@fgu.cas.cz (M.T.); 2 Department of Physiology, Faculty of Science, Charles University, Viničná 7, CZ 128 44 Prague 2, Czech Republic; 3 Laboratory of Biotransformation, Institute of Microbiology of the Czech Academy of Sciences, Vídeňská 1083, CZ 142 20 Prague 4, Czech Republic; miluse.vlachova@biomed.cas.cz (M.V.); slamova@biomed.cas.cz (K.S.); kren@biomed.cas.cz (V.K.); 4 Department of Health Care Disciplines and Population Protection, Faculty of Biomedical Engineering, Czech Technical University in Prague, Nám. Sítná, CZ 272 01 Kladno, Czech Republic

**Keywords:** galectin, HUVEC, ADSC, integrin, carbohydrate

## Abstract

Galectin-3 (Gal-3) is a β-galactoside-binding protein that influences various cell functions, including cell adhesion. We focused on the role of Gal-3 as an extracellular ligand mediating cell-matrix adhesion. We used human adipose tissue-derived stem cells and human umbilical vein endothelial cells that are promising for vascular tissue engineering. We found that these cells naturally contained Gal-3 on their surface and inside the cells. Moreover, they were able to associate with exogenous Gal-3 added to the culture medium. This association was reduced with a β-galactoside LacdiNAc (GalNAcβ1,4GlcNAc), a selective ligand of Gal-3, which binds to the carbohydrate recognition domain (CRD) in the Gal-3 molecule. This ligand was also able to detach Gal-3 newly associated with cells but not Gal-3 naturally present on cells. In addition, Gal-3 preadsorbed on plastic surfaces acted as an adhesion ligand for both cell types, and the cell adhesion was resistant to blocking with LacdiNAc. This result suggests that the adhesion was mediated by a binding site different from the CRD. The blocking of integrin adhesion receptors on cells with specific antibodies revealed that the cell adhesion to the preadsorbed Gal-3 was mediated, at least partially, by β1 and αV integrins—namely α5β1, αVβ3, and αVβ1 integrins.

## 1. Introduction

Galectins are β-galactosyl-binding proteins, which belong to lectins, i.e., carbohydrate-binding (glyco)proteins, interacting specifically with carbohydrate groups on other molecules, including those on the cell surface. Galectins were discovered in mammals, birds, amphibians, fish, nematodes, sponges, and also, in fungi. In mammals, sixteen types of galectins were found. Galectin-1, -2, -3, -4, -7, -8, -9, -10, -12, -13, -14, and -16 were identified in humans; galectin-5 and -6 were found in rats and mice; and galectin-11, -14, and -15 were found in ruminants [1,2,3,4,5].

Galectins are multifunctional pleiotropic molecules with a wide range of physiological and pathophysiological effects on cells, tissues, and organs. For example, galectins are involved in cell apoptosis, e.g., apoptosis of T cells (galectin-1 and -2); in cell–cell and cell–matrix adhesion (galectin-3 and -8); in cell proliferation (galectin-3); in inflammatory diseases, e.g., Crohn’s disease and ulcerative colitis (galectin-4); in cell differentiation, e.g., the differentiation of keratinocytes, and in the regeneration of epidermis and cornea (galectin-7) or the differentiation of adipocytes (galectin-12). Galectin-9 is involved in the defense against tuberculosis, and also in rheumatoid arthritis and metabolic disorders like diabetes. Galectin-10 is expressed in eosinophils and basophils and is involved in asthma. Galectins 13, -14, and -16 are expressed in the placenta and are supposed to be important for pregnancy tolerance development [1,2,3,4,5,6,7,8].

This study is focused on the role of galectin-3 (Gal-3) in cell–matrix adhesion. Gal-3 is present in many cell types, such as vascular, bone, adipose, fibroblast, and tumor cells, and it is involved in cell–cell and cell–matrix adhesion, cell proliferation, differentiation, phenotypic modulation, and also in cardiovascular diseases, tumor metastasis, and tumor vascularization. Although Gal-3 is involved in inflammation [9], venous thromboembolism [10], or cancer progression [3,7,11,12,13], it has also been reported to have many positive effects. These effects include, e.g., re-epithelialization of corneal, intestinal, and skin wounds (for review, see [14]), or a protective role in the uptake and removal of modified lipoproteins, accompanied with the downregulation of the proinflammatory pathways responsible for the initiation and progression of atherosclerosis [15]. Besides, the binding of Gal-3 to galactoside-capped ligands on endothelial progenitor cells (EPCs) promotes differentiation of these cells into mature endothelial cells and endothelial tube formation [16].

Gal-3 binds not only β-galactoside-capped glycans displayed on the surface of glycoproteins, but it can also bind synthetic carbohydrates and glycomimetics, which can be produced in relatively large quantities. The simplest ligands are represented by disaccharides LacNAc (Galβ1,4GlcNAc), LacdiNAc (GalNAcβ1,4GlcNAc), or thiodigalactoside (Galβ1,1-S-Gal), which can be attached to various biocompatible carriers [17,18] or derivatized [19,20]. In earlier studies, poly-LacNAc-based oligosaccharide ligands were bound, e.g., to a scaffold of bovine serum albumin [21], or LacdiNAc was attached to hydrophilic *N*-(2-hydroxypropyl) methacrylamide (HPMA) copolymers and used in the form of a glyconanomaterial [11]. These ligand–biomaterial complexes are promising for diagnostic and therapeutic purposes, e.g., for the selective recognition of Gal-3 both on the cell surface and in blood serum, and for the treatment of various disorders, particularly cancer [13].

In addition to cellular localization, Gal-3 is also secreted from cells to the extracellular environment, e.g., into the extracellular matrix (ECM), interstitial fluid, or circulation [13]. In the extracellular space, Gal-3 can act as an adhesion ligand for cells. It has been reported that the cells can bind Gal-3 with their integrin adhesion receptors, e.g., with some integrins with the β1 chain; with other transmembrane molecules, such as CD7, CD43, and CD45; and with glycoproteins associated with the cell membrane, such as fibronectin, vitronectin, and laminin [1,2,3]. From this point of view, Gal-3 ligands, such as LacNAc and LacdiNAc, as well as Gal-3 molecules themselves, could be attached to various biomaterials to colonize them with cells, e.g., for the purpose of tissue engineering.

However, in the context of possible biomaterial functionalization, it should be considered that Gal-3 can play a dual role in cell–matrix adhesion. Gal-3, and also other galectins, such as galectin-1 and galectin-8, can act as either positive or negative modulators of cell–matrix adhesion [1,3,6]. On the one hand, Gal-3 can enhance the cell–matrix adhesion by direct binding to integrin–adhesion receptors on the cells and by promoting the clustering of these receptors into focal adhesion plaques, which is a prerequisite of functional cell adhesion. On the other hand, Gal-3 can mediate the endocytosis of integrins, and thus their lower availability for cell adhesion. In addition, Gal-3 can weaken the cell–matrix adhesion by steric hindrance, when it is bound either to integrins or their ECM ligands [1,3]. The dual role of Gal-3 (and also other galectins) in cell adhesion has not yet been fully elucidated. It may depend on the cell type, type of integrin receptor, and, particularly, on the Gal-3 concentration. Moderate sub-micromolar concentrations of Gal-3 inhibited the adhesion of fibroblasts and tumor cells, while higher concentrations stimulated it [1]. Moreover, the form of galectin either as a soluble ligand or immobilized on the cell adhesion substrate also matters [6].

This study aims to systematically investigate the behavior of Gal-3 in cell–matrix adhesion as an extracellular adhesion ligand for cells, adsorbed on a cultivation substrate in a wide concentration range from 0.1 to 33 μM. We used human adipose tissue-derived stem cells (ADSCs) and human umbilical vascular endothelial cells (HUVECs) as the cell models. These two cell types were selected due to their importance in the tissue engineering of blood vessels. It is known that Gal-3 is expressed in immature cell types, such as endothelial progenitor cells [22] and mesenchymal stem cells [23], including ADSCs, from which it can be secreted into the extracellular space and can influence the adhesion and growth of other cell types [24]. In HUVEC cells in vitro, Gal-3 induced the formation of tubular capillary-like structures, which was associated with an increased expression of αVβ3 integrins on these cells [16].

**Scheme 1:**
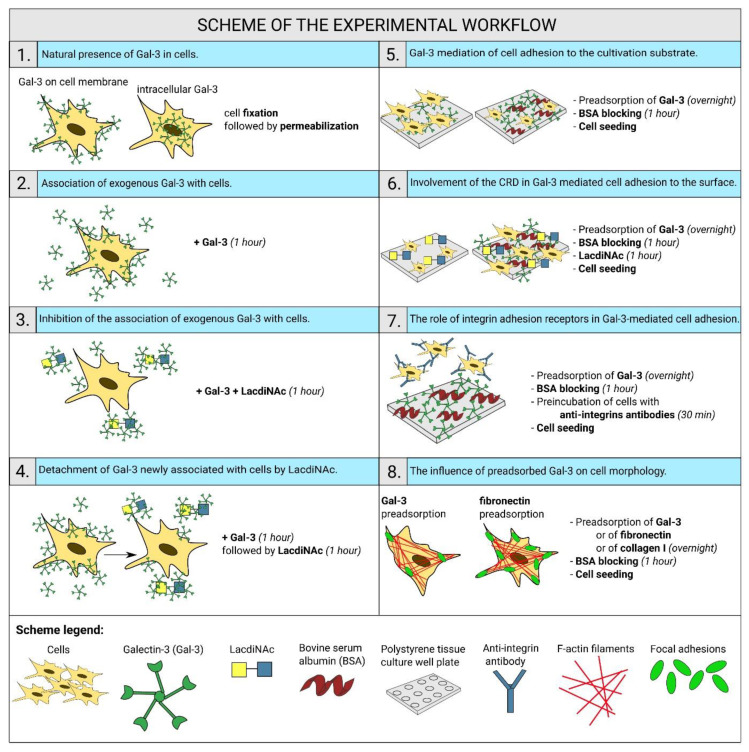
Summary of the experimental workflow with its main findings.

First, we verified the natural presence of Gal-3 in the used cell types, and then, we investigated (i) the ability of exogenous Gal-3 added to the culture medium to associate with cells and (ii) the ability of Gal-3 immobilized on the cultivation substrate to mediate cell adhesion. We found that exogenous free Gal-3 was able to associate with cells, and this association was reduced with Gal-3 ligand LacdiNAc in a concentration-dependent manner, while, importantly, cell adhesion to the substrate-immobilized Gal-3 was insensitive to LacdiNAc. These results indicate that while the free Gal-3 binds cells through its carbohydrate-binding site on the CRD domain, the Gal-3 immobilized on the cultivation substrate binds cells by another yet not fully explored site on its molecule; apparently, this binding site interacts with β1 and αV integrins on the surface of the cells, such as α5β1, αVβ3, and αVβ1 integrins. A comprehensive summary of our experimental work with its main findings is shown in Scheme 1.

## 2. Results

### 2.1. Gal-3 Is Naturally Present in ADSCs and HUVECs

**Figure 1:**
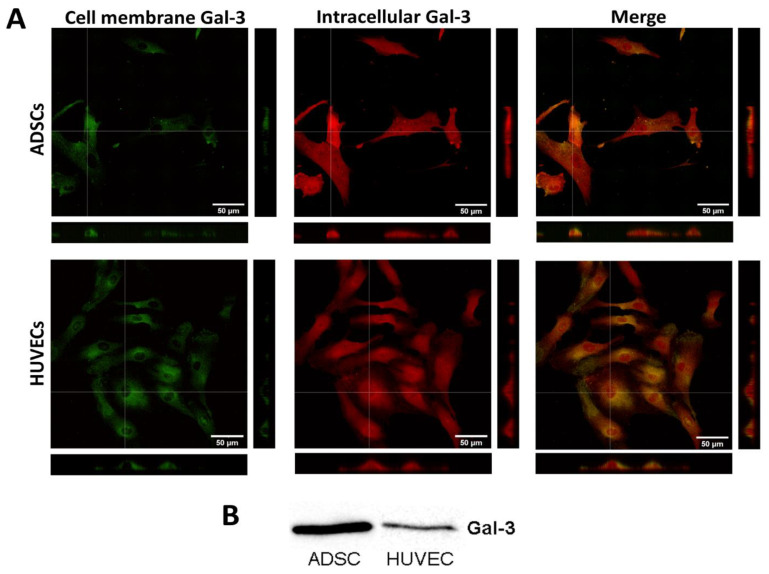
Localization of endogenous Gal-3 in ADSCs and HUVECs. (**A**) Immunofluorescence of Gal-3 in ADSCs and HUVECs in 1-day-old cultures. Cells were stained for Gal-3 present in the cell plasma membrane (green fluorescence) and for intracellular Gal-3 (red fluorescence). For Gal-3 staining, an anti-Gal-3 antibody produced in rabbits (Sigma-Aldrich, Cat. No. SAB4501746) was used. Orthogonal projections depict the localization of Gal-3 in the cell plasma membrane or the intracellular space. Andor Dragonfly 503 scanning disc confocal microscope; Zyla 4.2 PLUS sCMOS camera; objective HC PL APO 40×/1.10 W CORR CS2; scale bar 50 μm. (**B**) Western blot of Gal-3 expression in the cytosol of ADSCs and HUVECs. In both tested cell types, a Gal-3 band was detected at a size of 28 kDa.

As a starting point in this study, it was vital to check whether Gal-3 is present in our ADSCs and HUVECs. In order to distinguish the distribution of Gal-3 in the cell membrane and in the cytoplasm, the cells were first fixed, and without permeabilization, they were stained with an anti-Gal-3 antibody. Thus, this antibody bound only the Gal-3 present in the cell membrane (Figure 1A, green fluorescence). Then, the cells were permeabilized and incubated with an anti-Gal-3 antibody to visualize the intracellular Gal-3 (red fluorescence). The microphotographs show that Gal-3 is localized homogeneously in the cell membrane but to a lesser extent than in the cytoplasm. In the cytoplasm, Gal-3 is homogeneously distributed throughout the cells, with clear accumulation in the region of the cell nuclei. The cytosolic fraction of Gal-3 was further quantified by the Western blot method. The cytosolic Gal-3 expression detected by Western blotting is lower in HUVECs than in ADSCs (Figure 1B).

### 2.2. Exogenous Gal-3 Associates with ADSCs and HUVECs

**Figure 2:**
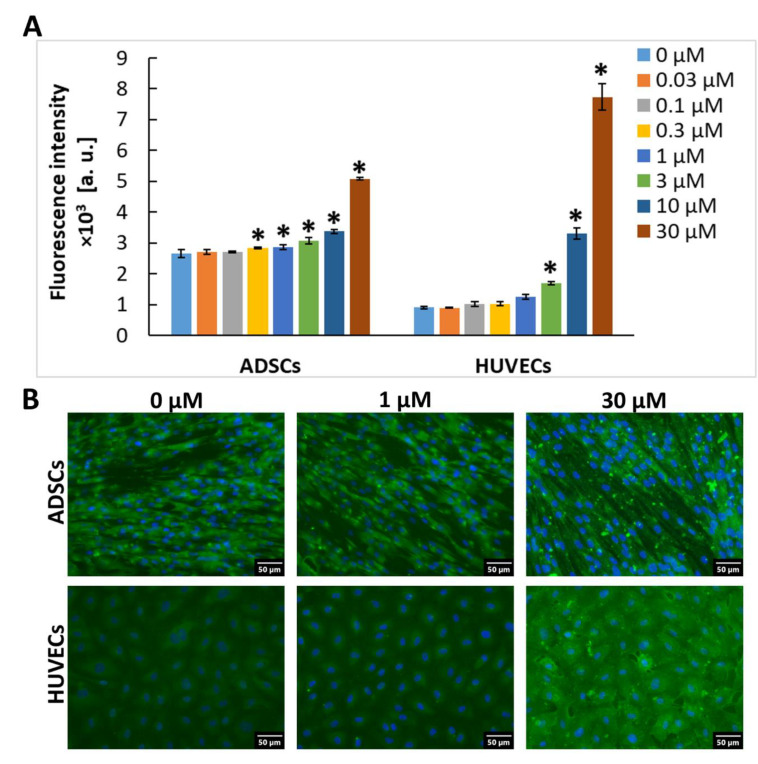
Association of exogenous Gal-3 with cells. For Gal-3 staining, the anti-Gal-3 antibody [EP2775Y] produced in rabbits (Abcam, Cambridge, UK; Cat. No. ab76245) was used. (**A**) The intensity of the fluorescence signal in wells with ADSCs and HUVECs after 1 h of incubation in a cultivation medium containing Gal-3 in a concentration range from 0 to 30 μM. The data are presented as the mean ± SD from 3 wells. Holm-Sidak test, *p* ≤ 0.05. The samples were statistically compared within the group of the indicated cell type. * Statistically significant difference in comparison with the control sample without the added Gal-3 (0 µM). (**B**) Representative images of immunofluorescence of Gal-3 in ADSCs and HUVECs after 1 h of incubation in a pure cultivation medium without Gal-3 (0 μM) and in the medium with 1 μM or 30 μM Gal-3. The cell nuclei were counterstained with Hoechst 33258. Olympus IX 71 microscope, DP 70 digital camera, obj. 20×, scale bar 50 μm.

Furthermore, we tested whether ADSCs and HUVECs can associate with Gal-3 molecules in the extracellular environment when it was present in the cell culture media. For this purpose, confluent cells in 96-well tissue culture plates were incubated for 1 h with various concentrations (0.03 to 30 µM) of Gal-3 diluted in the cell culture medium, and the membrane-bound Gal-3 was stained by immunofluorescence (after fixation with PFA and without the use of detergents, i.e., without permeabilization of the cell membrane). After staining, it was apparent that, in the galectin-free medium, ADSCs were more intensively stained than HUVECs, which suggests a higher basal content of Gal-3 in the cell membrane of ADSCs (Figure 2). A similar pattern was observed in permeabilized cells (Figure 1A,B), which implies that the basal content of Gal-3 was also higher inside the ADSCs. When exposed to Gal-3 in the culture medium, the intensity of fluorescence of Gal-3 in cells gradually increased with the increasing Gal-3 concentration, and this increase became statistically significant (in comparison with untreated cell samples) at 0.3 µM and 3 µM Gal-3 concentrations in ADSCs and HUVECs, respectively (Figure 2). At the 3 µM concentration, the intensity of the fluorescence of Gal-3 in ADSCs was still higher than in HUVECs. However, at the 10 µM concentration, the intensity of the fluorescence of Gal-3 was similar in ADSCs and HUVECs, and at 30 µM of Gal-3 in the cell culture medium, HUVECs showed a stronger ability to bind Gal-3, reaching a higher intensity of the fluorescence signal than ADSCs (Figure 2A).

### 2.3. The Association of Gal-3 with Cells Is Reduced by LacdiNAc

**Figure 3:**
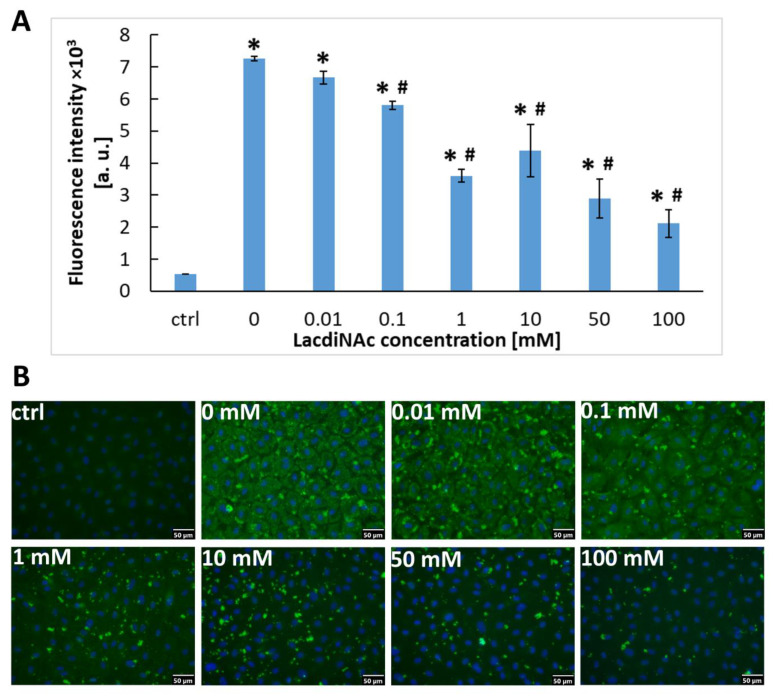
HUVECs after 1 h of incubation in a cultivation medium containing Gal-3 (30 μM) and various concentrations of LacdiNAc (0–100 mM). Control cells were incubated with a pure cultivation medium without Gal-3 and LacdiNAc. (**A**) Intensity of the fluorescence signal in HUVECs stained for Gal-3 (ex./em. = 485/528 nm). The data are shown as the mean ± SD from 3 wells. Holm-Sidak test, *p* ≤ 0.05. * Statistically significant difference in comparison with the control cells in the pure cultivation medium (ctrl). # Statistically significant difference in comparison with cells without LacdiNAc (0). (**B**) Immunofluorescence of Gal-3 in the cells compared in graph A. Anti-Gal-3 antibody (EP2775Y) produced in rabbits (Abcam, Cambridge, UK; Cat. No. ab76245) was used. The cell nuclei were counterstained with Hoechst 33258. Olympus IX 71 microscope, DP 70 digital camera, obj. 20×, scale bar 50 μm.

We aimed to reveal the mechanism of association of exogenous recombinant Gal-3 with the cell surface. We supposed that this association can be mediated by β-galactoside ligands on the cell surface, including the disaccharide LacdiNAc. This disaccharide is naturally present in a variety of glycoproteins in cells and acts as a specific ligand for the carbohydrate-binding site at the CRD domain of Gal-3 [25]. HUVECs were grown to confluence and incubated with a cultivation medium containing Gal-3 (30 μM) and various concentrations of LacdiNAc (0 to 100 mM), which were added to the medium 1 h before its exposure to the cells. The presence of LacdiNAc in the cultivation medium strongly decreased the ability of Gal-3 to bind to HUVECs in a concentration-dependent manner. Despite this, LacdiNAc was not capable of inhibiting the association of Gal-3 with the cells completely. Even at the highest (100 mM) concentration of LacdiNAc, the immunofluorescence of Gal-3 was still significantly higher than in control cells without exposure to Gal-3 (Figure 3A,B). Nevertheless, this result suggests that recombinant Gal-3 binds to β-galactoside ligands present on the cells by its CRD domain.

### 2.4. LacdiNAc Is Capable to Detach Gal-3 Newly Associated with the Cell Membrane

To further elucidate the exact mechanism of the inhibitory effect of LacdiNAc on the association of Gal-3 with the cells, we incubated confluent HUVECs with a cultivation medium containing either Gal-3 (30 μM) alone or LacdiNAc (1 mM) alone or both agents added either together or in a different order.

**Figure 4:**
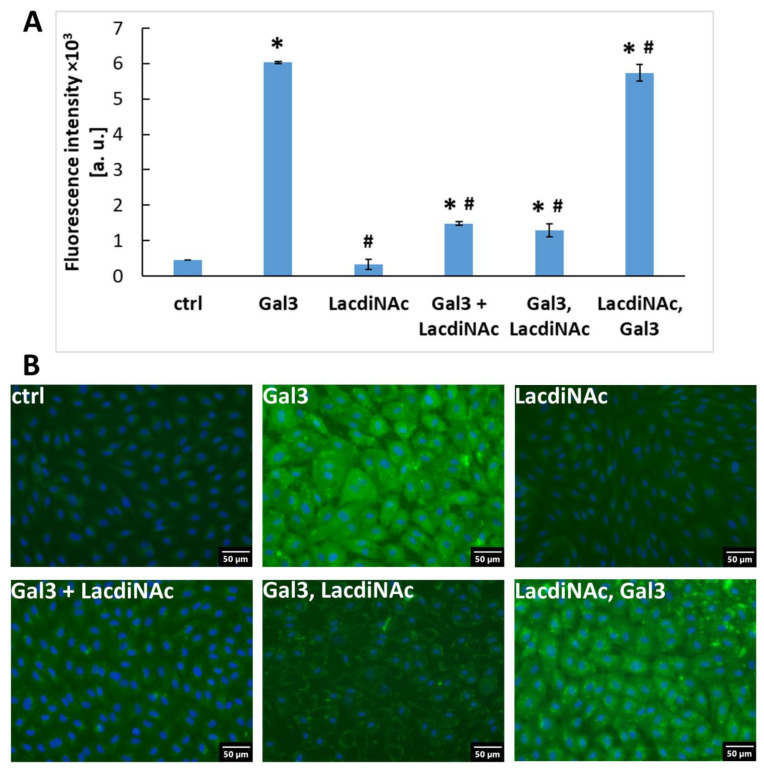
HUVECs after incubation in a cultivation medium containing Gal-3 (30 μM) or LacdiNAc (1 mM) or both molecules added together or in a different order. Control cells were incubated for 1 h in a pure cultivation medium. (**A**) Intensity of the fluorescence signal in HUVECs stained for Gal-3 (ex./em. = 485/528 nm). The data are shown as the mean ± SD from 3 wells. Holm-Sidak test, *p* ≤ 0.05. * Statistically significant difference in comparison with cells in the pure medium (ctrl), # Statistically significant difference in comparison with cells incubated with Gal-3 (Gal-3). (**B**) Immunofluorescence of Gal-3 in the cells compared in graph A. Anti-Gal-3 antibody (EP2775Y) produced in rabbits (Abcam, Cambridge, UK; Cat. No. ab76245) was used. The cell nuclei were counterstained with Hoechst 33258. Olympus IX 71 microscope, DP 70 digital camera, obj. 20×, scale bar 50 μm. Ctrl: cells in the pure medium; Gal3: cells incubated for 1 h in the medium with Gal-3; LacdiNAc: cells incubated for 1 h in the medium with 1-mM LacdiNAc; Gal3 + LacdiNAc: cells incubated for 1 h in the mixture of Gal-3 and LacdiNAc; LacdiNAc, Gal3: cells incubated for 1 h in the medium with LacdiNAc, then for 1 h in the medium with Gal-3; Gal3, LacdiNAc: cells incubated for 1 h in the medium with Gal-3, then for 1 h in the medium with LacdiNAc.

After the addition of Gal-3 into the cultivation medium for 1 h, Gal-3 was massively associated with the cells, as indicated by a markedly increased intensity of their immunofluorescence (Figure 4). When incubated for 1 h with LacdiNAc, the cells showed a similar presence of Gal-3 as in the control cells cultivated in the pure medium, which suggests that LacdiNAc was not capable of detaching Gal-3 naturally present on the plasma membrane. However, when the cells were incubated with a mixture of Gal-3 and LacdiNAc, the association of Gal-3 with the cells was markedly reduced, similar to the experiments described in Section 2.3. Moreover, when the cells were first incubated with Gal-3 for 1 h and then exposed to LacdiNAc for an additional 1 h, it was apparent that LacdiNAc was capable of detaching the newly associated recombinant Gal-3 from the cells. In contrast, when the cells were first treated for 1 h with LacdiNAc, then washed with PBS and incubated with Gal-3 for another 1 h, the fluorescence intensity remained comparable to the cells treated only with Gal-3 (Figure 4).

### 2.5. Gal-3 Mediates Cell Adhesion to the Cultivation Substrate

**Figure 5:**
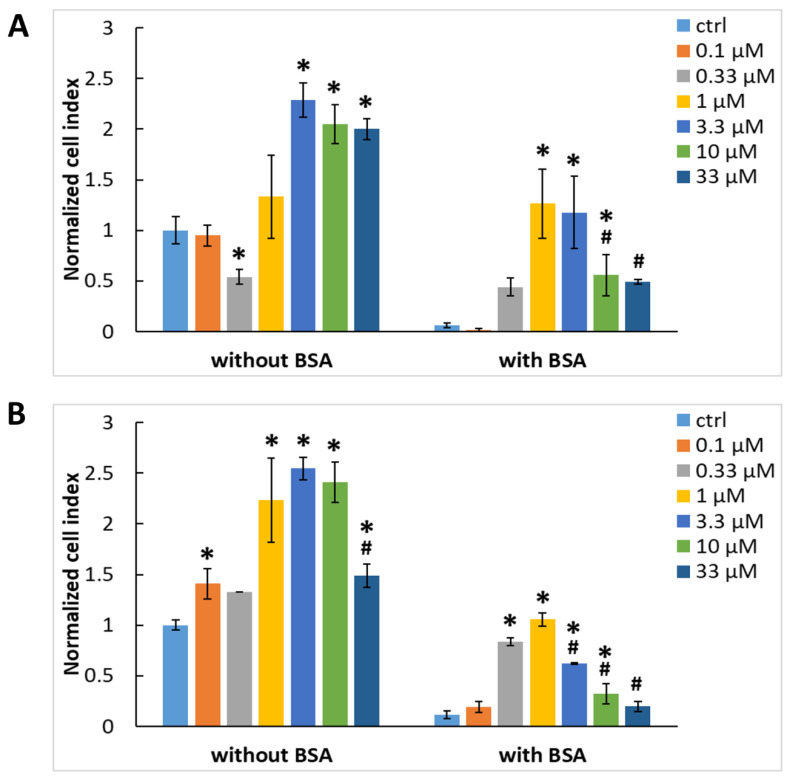
Initial adhesion of ADSCs (**A**) and HUVECs (**B**) to the wells of the E-plates in the xCELLigence system 1 h after seeding. The wells were adsorbed with various concentrations of Gal-3 (0.1–33 μM) either without blocking or with blocking using 0.5% *w/v* BSA. Cell index values were normalized to the control cell sample without adsorbed Gal-3 and BSA (ctrl without BSA). Mean ± SD (*n* = 3). Holm-Sidak test, *p* ≤ 0.05. The cell samples were statistically compared either within the group without BSA or within the group with BSA. * Statistically significant difference in comparison with the control samples without adsorbed Gal-3 (ctrl). # Statistically significant difference in comparison with the sample exhibiting the highest average value of the cell index.

We decided to adsorb Gal-3 on cultivation surfaces free of other adhesion ligands to test its ability to act as a mediator of adhesion of ADCSs or HUVECs to these surfaces. These surfaces were represented by plastic culture plates used, e.g., for ELISA. The evaluation of the cell-adhesive properties of Gal-3 was performed using an xCELLigence RTCA SP sensing device. The initial adhesion and spreading of ADSCs were measured in wells of E-plates adsorbed with various concentrations of Gal-3, ranging from 0.1 to 33 μM. The cells were incubated in DMEM without any additives to avoid the adsorption of cell adhesion-mediating molecules from the serum supplement, such as vitronectin and fibronectin or growth factors, which can also influence the cell adhesion [26]. The cell adhesion and spreading were monitored for four hours. One hour after cell seeding, i.e., after the time interval necessary for the initial adhesion of the cells, mediated by the interactions of the cell surface adhesion receptors (e.g., integrins) with ligands on the cultivation surface, Gal-3 significantly enhanced cell adhesion to the well bottoms in concentrations of 3.3 to 33 μM, as indicated by the increasing cell index (Figure 5A). However, in the negative control assay in the absence of Gal-3, the control wells allowed the initial adhesion to a similar extent as in lower concentrations of Gal-3, i.e., from 0.1 to 1 μM. Similar results were also obtained four hours after cell seeding, which is the time interval when the spreading of the adhered cells typically happens (Figure S1A, Supplementary Materials). This suggests nonspecific cell adhesion mediated by weak chemical bonds, e.g., electrostatic interactions, polar interactions, hydrogen bonding, van der Waals forces, etc., between the cell membrane and the well material [27]. In addition, some cell adhesion-mediating molecules, such as fibronectin, vitronectin, collagen, and laminin, can be retained on the cell surface even after trypsinization, which preceded cell seeding, and these molecules can also contribute to nonspecific cell adhesion. Therefore, the wells adsorbed with Gal-3 were blocked with 0.5% *w/v* BSA, a protein nonadhesive for cells, to prevent nonspecific interactions of cells with the well bottom surface (Figure 5A and Figure S1A). After blocking the nonspecific binding sites in the wells by BSA, the highest cell adhesion and spreading were observed at the 1 μM concentration of Gal-3, and the lower or higher concentrations were not optimal for cell adhesion mediated by Gal-3 (Figure 5A and Figure S1A). At the same time, the well bottoms without Gal-3 or with its lowest concentration (0.1 μM) did not promote almost any cell adhesion or any cell spreading.

The same set of experiments was then performed with HUVECs (Figure 5B and Figure S1B), and the adhesion behavior of these cells was compared with that of ADSCs. Interestingly, ADSCs and HUVECs showed a comparable affinity to Gal-3, which is in contrast to our previous results (Figure 2), where the binding of Gal-3 from the culture medium to the cell surface appeared to be higher in HUVECs. On the other hand, a higher association of Gal-3 with HUVECs occurred only at the 30 µM concentration of Gal-3, while at lower concentrations, this association was similar (10 µM) or even lower (0.03-3 µM) in comparison with ADSCs (Figure 2). Nonetheless, all these results are promising, because they suggest that Gal-3 acts as an adhesive molecule, and it could be further used for coating biomaterials, e.g., the inner surface of artificial vascular grafts, to enhance the adhesion of endothelial cells or their potential precursors in the form of stem cells, and thus to promote the formation of a confluent endothelial cell layer.

### 2.6. Gal-3-Mediated Cell Adhesion to the Surface Does Not Involve the CRD-Domain

**Figure 6:**
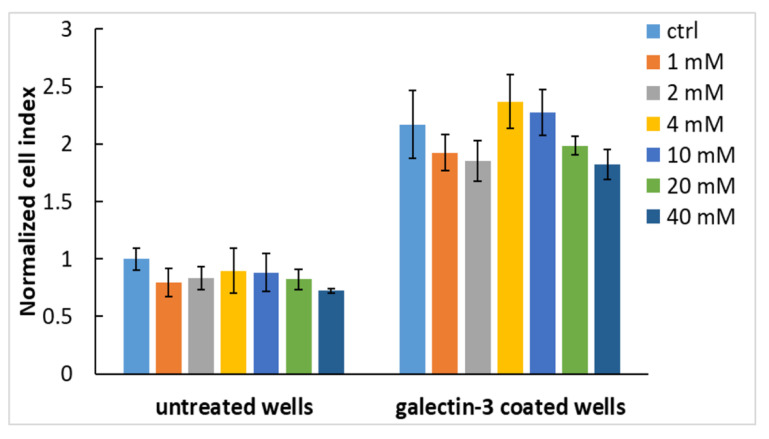
The effect of LacdiNAc on the initial adhesion of ADSCs one hour after seeding into untreated wells or wells preadsorbed with 1 μM Gal-3 and blocked with BSA. Before cell seeding, both groups of wells were preincubated with LacdiNAc in concentrations from 1 to 40 mM. Wells without LacdiNAc served as control samples (ctrl). Cell index values were normalized to the sample without Gal-3 adsorption and without LacdiNAc in the medium (ctrl - untreated wells). Mean ± SD from 3 wells. Holm-Sidak test, *p* ≤ 0.05. The cell samples were statistically compared either within the group of untreated wells or within the group of Gal-3-adsorbed wells. * Statistically significant difference in comparison with the control cells (ctrl).

Our experiments then continued with testing the potential inhibitory activity of LacdiNAc, a β-galactoside ligand for Gal-3, on Gal-3-mediated cell adhesion. Wells in the xCELLigence E-plates were adsorbed with 1 μM Gal-3, blocked with 0.5% *v*/*w* BSA, and preincubated with various concentrations of LacdiNAc (1 to 40 mM) before cell seeding. The results showed that LacdiNAc did not inhibit the initial adhesion of ADSCs to the Gal-3-adsorbed well bottoms one hour after seeding (Figure 6) or the cell spreading after four hours of incubation (Figure S2), except for the highest concentration of LacdiNAc, which slightly but significantly decreased the cell adhesion to the wells preadsorbed with Gal-3.

The effect of LacdiNAc on nonspecific adhesion (i.e., adhesion not mediated by Gal-3) was also evaluated with ADSCs cells. The results showed that, similarly, LacdiNAc did not considerably affect the initial cell adhesion and spreading on the well bottoms non-adsorbed with Gal-3 (Figure 6 and Figure S2).

### 2.7. Integrins Are Cell Receptors for the Substrate-Bound Galectin

**Figure 7:**
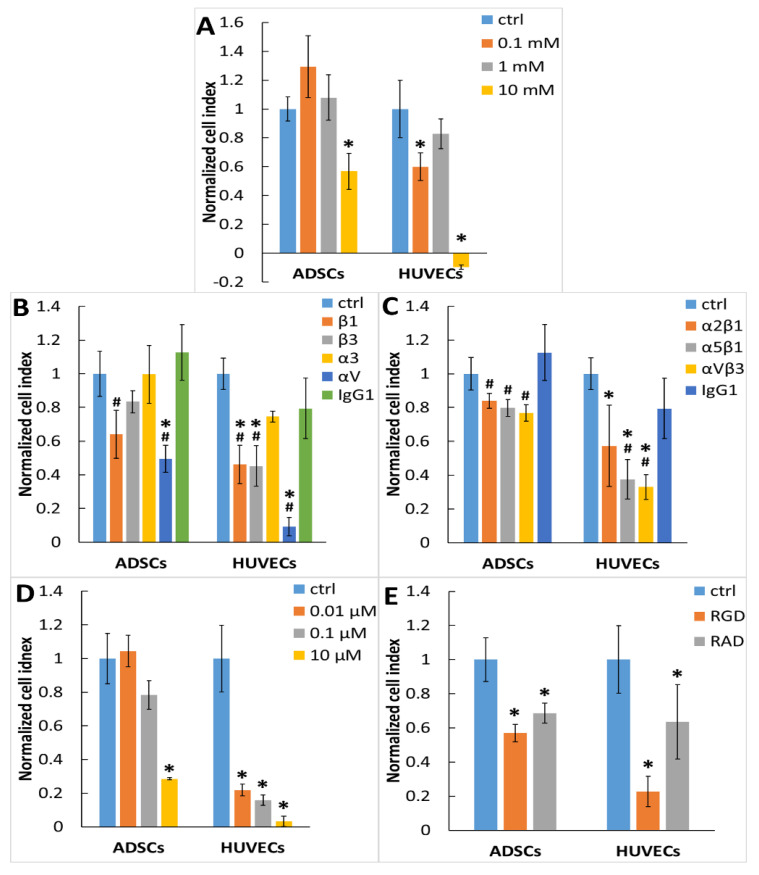
Role of integrins in the initial adhesion of ADSCs and HUVECs to substrate-immobilized Gal-3 (1 h after seeding). (**A**) The cells were seeded in a medium containing EDTA in concentrations ranging from 0.1 to 10 mM. (**B**) The cells were seeded in the medium containing antibodies against the β1, β3, α3, and αV integrin subunits. The antibody against αV integrin subunit was used in a 1:25 dilution, and the other antibodies were used in a concentration of 20 μg/mL. Nonspecific mouse IgG1 was used as an isotype control. (**C**) The cells were seeded in the medium containing antibodies against α2β1, α5β1, or αVβ3 integrin receptors. Nonspecific mouse IgG1 was used as an isotype control. Antibodies were used in a concentration of 20 μg/mL. (**D**) The cells were seeded in a medium containing the αVβ1 inhibitor in concentrations ranging from 0.01 to 10 µM. (**E**) The cells were seeded in a medium containing the GRGDSP peptide. The GRADSP peptide was used as a negative control. Both peptides were used in a concentration of 200 µM. Before cell seeding, the wells were coated with 1 µM Gal-3 and blocked with 0.5% *w/v* BSA. The cell index values were normalized to the untreated sample in a pure medium without any additive (ctrl). Mean ± SD from 3 wells. Holm-Sidak test, *p* ≤ 0.05. The samples were statistically compared within the group of the indicated cell type. * Statistically significant difference in comparison with the control cells (ctrl). # Statistically significant difference in comparison with the cells incubated with the isotype control (IgG1).

Our experiments continued with searching for receptors on the cell surface that are responsible for binding to Gal-3 preadsorbed on the cell surface. It is generally known that cell adhesion is mainly mediated by integrin receptors, which are heterodimeric transmembrane proteins formed by α and β subunits, and that these receptors are also involved in the galectin-mediated cell-matrix adhesion [1,2,3]. Here, we show that the highest concentrations of EDTA (10 mM), a potent calcium chelator and a well-known inhibitor of integrin receptors, inhibited the initial cell adhesion and spreading on Gal-3-coated surfaces (Figure 7A and Figure S3A). These results indicate the involvement of integrins in Gal-3-mediated adhesion of both studied cell types because calcium is essential for the adhesion function of integrins.

To further specify the involvement of particular integrin types in Gal-3-mediated cell adhesion, we preincubated the cells with various blocking antibodies against the integrin subunits, and then we seeded the cells on Gal-3-coated surfaces. We used antibodies against the integrin subunits most commonly involved in the cell–matrix interaction and expressed by a wide range of cell types—namely, the β1, β3, and αV subunits—and also an antibody against the α3 integrin subunit. The latter is a component of the α3β1 integrin, a promiscuous receptor expressed mainly by endothelial cells, mediating the adhesion to laminin, fibronectin, or collagen [28]. As indicated by flow cytometry, these integrin subunits were also present in the ADSCs and HUVECs used in our study (Figures S4 and S5 in the Supplementary Materials). Our results showed that the adhesion of ADSCs was strongly blocked both with the αV and β1 integrin antibodies but only slightly with the β3 integrin antibody whereas the adhesion of HUVECs was significantly inhibited by all three antibodies (Figure 7B). The inhibition of the cell spreading four hours after seeding of both cell types was more pronounced with the αV and β1 integrin antibodies (Figure S3B). In contrast, the adhesion of ADSCs was not blocked with the α3 integrin antibody. This is in line with the results from the flow cytometry analysis, which showed only a weak expression of the α3 integrin subunit in ADSCs. In contrast, the adhesion of HUVECs was partially but significantly blocked with the α3 integrin antibody, mainly 4 h after cell seeding (Figure S3B).

Integrin receptors can be divided into specific groups according to their affinity to various ECM proteins or various amino acid sequences within these proteins. There are RGD-binding integrins recognizing fibronectin (e.g., α5β1) or vitronectin (e.g., αVβ3), collagen-binding receptors (e.g., α1β1 or α2β1), or laminin-binding receptors (e.g., α6β1 or α7β1 [29]). Since the laminin-binding receptors α6β1 or α7β1 are weakly expressed in ADSCs [30], we decided to test the most widespread receptors from the remaining groups. For this aim, we used antibodies against the α2β1 integrin (collagen I receptor), and also against the α5β1 and αVβ3 integrins, which are receptors for fibronectin and vitronectin. These two blood serum-derived proteins are the main proteins mediating cell–substrate adhesion in vitro. Thus, it may be supposed that integrin receptors for fibronectin and vitronectin would be highly expressed in cultured cells. As proven by flow cytometry, the α2β1, α5β1, and αVβ3 integrins were highly expressed in ADSCs and HUVECs in our study (Figures S4 and S5 in the Supplementary Materials). Apparently, the α2β1, α5β1, and αVβ3 integrin antibodies had only a slightly negative influence on the adhesion of ADSCs. On the other hand, the inhibitory effect of these antibodies on the adhesion of HUVECs was more pronounced. Four hours after seeding, both cell types showed the lowest cell index values after blocking with the α5β1 and αVβ3 integrin antibodies (Figure S3C). However, the reduction of the cell index values was relatively small, especially in the αVβ3 antibody. This suggests that α5β1 and αVβ3 were not the only receptors responsible for recognizing the substrate-bound Gal-3. From the previous results (Figure 7B), it is obvious that the adhesion to Gal-3 is inhibited mainly by the αV and β1 integrin subunits. This led us to the hypothesis that the αVβ1 integrin could be another receptor with affinity to Gal-3. Since there is no commercially available antibody specific to the αVβ1 integrin, we tested a novel peptide inhibitor of the αVβ1 integrin, which was shown to suppress the αVβ1 integrin-mediated adhesion of fibroblasts in nanomolar concentrations, and also to attenuate the experimentally induced lung and liver fibrosis in mice [31]. When applied in concentrations of 0.01, 0.1, and 10 μM, the inhibitor significantly decreased the Gal-3-mediated adhesion and spreading in HUVECs in a concentration-dependent manner (Figure 7D and Figure S3D). It is tempting to say that the cell adhesion to Gal-3 is mediated mainly by the αVβ1 integrin; however, the adhesion of ADSCs was significantly blocked only by the 10 μM concentration of the inhibitor. Moreover, at the 10 μM concentration, the adhesion of ADSCs was also blocked at comparable levels in collagen I, vitronectin, or fibronectin-coated wells (Figure S6 in the Supplementary Materials). In addition, this integrin inhibitor has also been shown to interact with other RGD-binding integrins (e.g., αVβ3, α5β1, or α8β1) when applied at micromolar concentrations [32]. The possible role of RGD-binding integrins in Gal-3-mediated cell adhesion was further confirmed by incubating the cells with the RGD-containing peptide. It was found that the cell adhesion and spreading were decreased by the RGD peptide in both ADSCs and HUVECs (Figure 7E and Figure S3E). Interestingly, the cell adhesion to substrate-bound Gal-3 was also partially inhibited with a peptide-containing RAD sequence, which was supposed to serve as a negative control.

### 2.8. Cell Morphology on the Surface Preadsorbed with Gal-3

To visualize the morphology of the cells adhered to the Gal-3-coated surface, we adsorbed Gal-3 on the 96-well glass plate and blocked the nonspecific binding sites with BSA. The reference wells were either left uncoated or coated with fibronectin or collagen I and blocked with BSA or only blocked with BSA. The cells were fixed four hours after seeding into the pure medium. Changes in the cell morphology on various adhesion substrates were also quantified by evaluation of the cell morphology characteristics (i.e., cell area, perimeter, circularity, and solidity; Figure S9 in the Supplementary Materials).

ADSCs on Gal-3 showed similar morphology characteristics to the cells on pristine, uncoated glass with a slightly better-developed actin cytoskeleton (Figures S7A, S8A, and S9 in the Supplementary Materials). ADSCs on fibronectin were more spread out (Figure S9A,B), with visible vinculin-containing focal adhesions located at the cell periphery. The cells on collagen I showed a lower amount of focal adhesions than on fibronectin. Blocking the pristine glass surface with BSA prevented the ADSCs from spreading adequately and forming a well-developed actin cytoskeleton.

HUVECs on the control pristine glass displayed more rounded morphology than ADSCs (Figure S9C, a relatively high circularity parameter), but they were still relatively well-spread with the actin cytoskeleton and focal adhesions at the cell periphery (Figures S7B and S8B in the Supplementary Materials). HUVECs seeded on Gal-3 formed clusters of cells. In contrast to the relatively well-spread ADSCs on the Gal-3-coated surface, HUVECs displayed a stellate-like morphology with a poorly developed actin cytoskeleton, located mainly in the cell protrusions, which was also manifested in a smaller average cell spreading area, a shorter cell perimeter, and also in a relatively low solidity, i.e., a high occurrence of cell protrusions (Figure S9). HUVECs on fibronectin-coated glass showed well-formed vinculin-containing focal adhesions homogeneously distributed throughout the entire substrate-contacting cell membrane and a strong actin cytoskeleton. HUVECs on collagen I displayed a well-developed cytoskeleton, but the cytoskeletal structure was slightly worse than the one on fibronectin. The cells on pristine glass blocked with BSA were poorly spread out and were not able to form almost any cytoskeletal structures (Figures S7B, S8B and S9 in the Supplementary Materials).

## 3. Discussion

The aim of this work was to investigate Gal-3 as an adhesion receptor on the cell surface and as an extracellular ligand binding to the cell surface receptors. Our results show that ADSCs express higher amounts of Gal-3 than HUVECs. It is in accordance with earlier findings that stem cells and other immature cell types often contain a higher amount of Gal-3 than more mature cells [22,33]. However, when exposed to Gal-3 in the cell culture medium, HUVECs reached a considerably higher intensity of fluorescence signal than ADSCs (Figure 2). It could therefore be concluded that HUVECs showed a stronger ability to bind Gal-3 than ADSCs, at least at higher Gal-3 concentrations in the extracellular environment. Gal-3 interacts with the cell surface receptors mainly by binding to glycans on proteins [34], and its binding activity depends on the glycosylation pattern on the cell surface [35,36]. The binding of Gal-3 to the cell surface glycoproteins is mediated by terminal β-galactoside groups of *N*-glycans [35], and it is negatively regulated by sialylation [35,37]. Supposedly, ADSCs contain lower amounts of β-galactoside groups available for Gal-3 binding compared to HUVECs. Thus, the difference between Gal-3 binding to ADSCs and HUVECs can be explained by different glycosylation patterns in their cell surface glycoproteins. Our preliminary experiment with tunicamycin (an inhibitor of *N*-glycosylation) showed that the association of Gal-3 with the cell surface of ADSCs is independent of the tunicamycin treatment (up to 10 μg/mL, which was already detrimental to the cells; data not shown). In future experiments, it would be interesting to treat the cells with other glycosylation inhibitors (e.g., swainsonine or benzyl *N*-acetyl-α-d-galactosaminide) to obtain a deeper insight into the influence of glycosylation on the association of Gal-3 with the cell plasma membrane.

In addition, ADSCs and HUVECs may differ in the further processing of Gal-3 after its binding to the cell surface. After binding to cells, Gal-3 is rapidly internalized by endocytosis, which is either carbohydrate-dependent (as in nonphagocytic cells) or independent (as in cells capable of phagocytosis; [38]). After endocytosis, some Gal-3 molecules are trafficked into various intracellular compartments, including lysosomes, while some of them are recycled on the cell membrane [39]. Galectins can also counteract the endocytic uptake by forming lattices on the plasma membrane, which depends also on the concentration and structure of the glycoprotein and glycolipid Gal-3 ligands [40].

Gal-3 (28 kDa) consists of an N-terminal collagen-like domain that enables protein oligomerization and a C-terminal domain (carbohydrate recognition domain; CRD) binding β-galactosides [41]. We found that Gal-3 binds to the surface of HUVECs in a manner inhibitable by carbohydrates, such as LacdiNAc. LacdiNAc is a β-galactoside naturally present in a variety of glycoproteins that acts as a specific ligand for Gal-3 [17,21,25]. Naturally occurring β-galactosides (e.g., lactose) have also been shown to inhibit the binding of Gal-3 to the cell surface [34], as well as inhibit the subsequent biological effects triggered by Gal-3 [4,34]. Besides, there is a broad variety of other carbohydrate-based Gal-3 inhibitors [4,13,20,42,43].

The β-galactoside LacdiNAc, chosen for this study, can bind not only to Gal-3 but, to some extent, also to other proteins from the galectin family (e.g., Gal-2 or -7; [44]). On the other hand, LacdiNAc has a low binding affinity to Gal-1 or Gal-4 [25,44]. Of the twelve types of human galectins identified so far [5], only four types of galectin are expressed abundantly (e.g., Gal-1, -3, -8, and -9) in HUVECs, while the expression of the other galectins is only faint or undetectable [45]. A similar expression pattern was also observed in mesenchymal stem cells [46]. Therefore, in our experimental setup where excess amounts of exogenous Gal-3 were applied, we can consider any possible interaction of LacdiNAc with other proteins from the galectin family insignificant.

Our study also revealed that even the highest concentration of LacdiNAc (100 mM) did not stop the association of recombinant Gal-3 with the plasma membrane of HUVECs completely. This finding correlates with a study where the treatment of the plasma membrane with lactose led to the detachment only of a small part of membrane-associated Gal-3 [47]. This phenomenon was also observed in other studies [16,34,40,44], and it should be taken into account when LacdiNAc is used therapeutically in Gal-3-overexpressing tissues [11]. We hypothesized that the inability to detach all membrane-associated Gal-3 with the LacdiNAc ligand is caused either by the presence of high-affinity binding sites for Gal-3 on the cell surface by the involvement of a noncanonical binding site on Gal-3 CRD [48,49,50] or, possibly, by the presence of another binding site on the Gal-3 molecule. The two binding sites on Gal-3 CRD (canonical and noncanonical) can be occupied by the ligands at the same time.

Our results showed that Gal-3 optimally enhanced the cell adhesion when adsorbed to the plastic surface in the concentration of 1 μM. Previous research showed both inhibitory and enhancing effects of Gal-3 in the culture medium on the cell adhesion to a cultivation substrate [1,51]. These contradictory results could be explained by the ability of Gal-3 to form oligomeric structures. When present in a solution at lower concentrations, Gal-3 stays in its monomeric form and binds in a monovalent manner to the cell surface or extracellular matrix proteins, creating steric hindrance and lowering the strength of the cell adhesion. In higher concentrations, Gal-3 can form oligomeric complexes and binds to the cell surface and extracellular matrix glycoproteins simultaneously, functioning as an “adhesion glue” (or a crosslinking agent) and enhancing the cell adhesion mediated by specific adhesion sequences on extracellular glycoproteins. When present in amounts exceeding the concentrations of the carbohydrate ligands on the extracellular matrix or cell surface, Gal-3 tends to bind the ligands in a monovalent manner, which leads to the inhibition of cell adhesion [1]. The concentration at which Gal-3 switches from its inhibitory to adhesion-enhancing activity and *vice versa* is micromolar [51]. Interestingly, our results showed that relatively high concentrations of adsorbed Gal-3 (i.e., 3–33 μM) were not optimal for cell adhesion. When adsorbed at high concentrations (tens of micromoles per liter) to the surface, Gal-3 was supposed to form multivalent oligomeric complexes [52]. However, we suggest that a high concentration of Gal-3 in the solution led to the formation of clustered Gal-3 molecules bound in a high density on the substrate, which caused steric inhibition of the correct interaction of Gal-3 with the adhesion receptors on the cell surface, thus decreasing the cell adhesion intensity. The negative effect of the excess of the adhesion ligands was observed before [53] with the RGD ligands. Our results showed that the adsorbed Gal-3 mediated the cell adhesion of both HUVECs and ADSCs. The adhesion could not be inhibited by LacdiNAc, which suggests the involvement of another cell adhesion-mediating mechanism than the interaction of the Gal-3 CRD canonical binding site with carbohydrates. It has been reported that the cells can bind Gal-3 in other alternative ways, such as by integrin adhesion receptors α_1_β_1_, α_3_β_1,_ and α_7_β_1_. Other cell membrane-associated molecules binding galectin include CD7 (an immunoglobulin found on thymocytes and mature T cells), CD43 (sialophorin), and CD45 (tyrosine phosphatase on hematopoietic cells), fibronectin, vitronectin, laminin, and also lysosome-associated membrane proteins LAMP-1 and LAMP-2 [1,2,3]. In the cited studies, Gal-3 was believed to interact with the carbohydrate components of all the mentioned molecules, but it is questionable whether this interaction is always mediated by the CRD canonical binding site in the Gal-3 molecule. Alternative binding molecules for Gal-3 on cell surfaces could also include proteins.

In the search for alternative binding sites for Gal-3 on the cell surface, we concentrated on the integrin adhesion receptors on cells, which have been shown to be involved in Gal-3-mediated cell–matrix adhesion [1,2,3]. Integrin adhesion receptors comprise at least 18 α- and 8 β-subunits, which can assemble into at least 24 unique integrin receptors [29]. Thus, we used antibodies against the mentioned subunits, and also against the β3 subunit, which is a component of the so-called “vitronectin receptor”- the αVβ3 integrin, widely expressed in immature and proliferating cells, including the cells grown in conventional serum-supplemented cell culture systems. The mentioned integrin subunits are expressed in many cell types, including ADSCs and HUVECs, as shown by the flow cytometry analysis (Figures S4 and S5 in the Supplementary Materials) in our study, and also elsewhere [54,55,56]. Our results showed that cell adhesion was blocked by antibodies against the αV and β1 integrin subunits but not substantially by the anti-β3 subunit antibody.

Our observations that the cell adhesion to Gal-3 is mediated mainly by the integrin subunits αV and β1 are also in good correlation with the study by Zhuo et al. (2008) [37]. They observed a significant decrease in the adhesion of colonocytes to Gal-3 when incubated with an integrin β1 functional blocking antibody. Moreover, they found that the adhesion is negatively regulated by the sialylation of the β1 integrin subunit. The role of β1 integrin as a surface receptor for Gal-3 was also proven in a study by Fukumori et al., performed on T cells [34].

Our results further showed that the adhesion to substrate-bound Gal-3 is mediated, at least partially, by integrins α5β1 and αVβ3. These integrins are present on both ADSCs and HUVECs (Figures S4 and S5 in the Supplementary Materials). They are receptors for fibronectin and vitronectin, playing an important role in angiogenesis [57]. Another integrin involved in cell adhesion to substrate-bound Gal-3 was the αVβ1 integrin, but its involvement was more pronounced in HUVECs than in ADSCs, as indicated by the blocking experiments (Figure 7D). Interestingly, the involvement of the α5β1 and αVβ3 integrins and their subunits, such as β1, β3, and αV, was also more pronounced in HUVECs (Figure 7B,C), although the flow cytometry showed comparable amounts of this molecule in the HUVECs and ADSCs (Figures S4 and S5 in the Supplementary Materials). On the other hand, the α3 integrin subunit (representing the α3β1 integrin receptor), which is expressed mainly in HUVECs (Figures S4 and S5 in the Supplementary Materials), appeared to be involved in Gal-3-mediated adhesion to a lesser extent. These results suggest that the cell type is an important factor affecting the role of integrins in Gal-3-mediated cell adhesion. This phenomenon may deserve further investigation in the future.

The α5β1, αVβ3, and also αVβ1 integrins typically recognize the RGD motif within the ECM molecules. Therefore, it can be hypothesized that the adhesion of ADSCs and HUVECs to Gal-3 is mediated by a specific amino acid adhesion sequence in the Gal-3 structure, which binds to integrin receptors. This is in accordance with our results where the adhesion of the cells to Gal-3 was significantly blocked by the 10 μM αVβ1 inhibitor, which is of an oligopeptidic RGD-like nature. In micromolar concentrations, this inhibitor can block a wide range of RGD-binding integrins [32]. The RGD sequence itself can also block cell adhesion to Gal-3 [58]. Surprisingly, the adhesion of ADSCs and HUVECs was partially inhibited by the RGD-containing peptide (Figure 7E), albeit there was no RGD motif in Gal-3 primary structure. However, it is not uncommon in the integrin superfamily that a specific type of integrin can bind to different ECM ligands in a “promiscuous” manner and different types of integrins can bind to the same ligand [59]. Therefore, it cannot be excluded that some other amino acid sequence(s) in the Gal-3 molecule may take part in the integrin–Gal-3 interactions. Another possibility would be the involvement of a noncanonical binding site on Gal-3 CRD, which was shown to be independent of the blocking of the canonical binding site with β-galactosides [49].

When adhered to glass coated with Gal-3, ADSCs showed a similar morphology to the untreated glass. In contrast, HUVECs on the Gal-3-coated surface exhibited a distinct morphology when compared with the cells on fibronectin coating or with untreated glass. HUVECs formed clusters of cells with the poorly developed cytoskeleton and “stellate-like” morphology. Similarly, the formation of “stellate-like” morphology with numerous lamellipodia and filopodia was observed in epithelial cells after incubation with Gal-3 [60]. There, the Gal-3-induced changes in the cell morphology were mediated by the α3β1 integrin. In our study, the strong expression of the α3β1 integrin was observed in HUVECs; in contrast, only a mild expression of this integrin was detected in ADSCs. This would explain the differences in the cell morphology of these two cell types on the Gal-3-coated surface. All these results suggest that, although Gal-3 interacts with the integrin receptors and enhances cell adhesion, it is not able to trigger specific integrin-mediated cytoskeletal formation. It can be explained by the fact that Gal-3 is not a “classical” ligand for integrin receptors, such as specific oligopeptide sequences within ECM molecules (e.g., RGD) that bind both α and β chains within the integrin receptor [59]. As shown in our experiments, Gal-3 can bind only a single subunit of the integrin receptor, e.g., the αV subunit within the αVβ3 integrin, as documented in a relatively high inhibitory activity of the αV blockers and no activity of the β3 blockers. Such a partial binding cannot ensure a fully functional cell adhesion to Gal-3, at least in some cell types.

Our findings on cell–material adhesion mediated by the optimal concentration of Gal-3 adsorbed on the material surface may be applied in vascular tissue engineering. Gal-3 biofunctionalization of relatively bioinert materials used for vascular prostheses could lead to a better cell adhesion, as observed in the case of ADSCs and HUVECs in our in vitro experiments. The biofunctionalization of biomaterials with Gal-3 could be used as a novel alternative approach to the functionalization with short ECM-derived oligopeptides, which serve as ligands for integrin adhesion receptors and which have been widely investigated for the improved endothelialization of vascular replacements based on synthetic polymers [61,62] or decellularized matrices [63]. Furthermore, due to its positive role in angiogenesis [16], Gal-3 could also be utilized in broader tissue engineering applications. The Gal-3 biofunctionalization of 3D scaffolds (e.g., for bone or skin tissue engineering) could attract endothelial cells, facilitate their adhesion, potentiate capillary formation, and thus support the vascularization process of a scaffold that is very important for the long-term survival and proper functioning of the newly engineered tissues.

## 4. Materials and Methods

### 4.1. Preparation of Human Recombinant Gal-3

A recombinant His-tagged construct of Gal-3 was produced and purified as described previously [21]. Gal-3 was recombinantly expressed in the cells of *E. coli* Rosetta 2 (DE3) pLysS. The cultivation of precultures (60 mL in 0.5 L baffled flasks) was done overnight in Lysogeny broth (LB) medium (0.5% *w/v* yeast extract, 1.0% *w/v* tryptone, and 0.5% *w/v* NaCl; pH 7.4) at 220 rpm and 37 °C. The medium contained ampicillin (100 mg/L) and chloramphenicol (34 mg/L). After 17 h, the main cultures (600 mL in 3 L baffled flasks) in Terrific broth (TB) medium (2.4% *w/v* yeast extract, 1.2% *w/v* tryptone, 0.4% *v/v* glycerin, 17 mM KH_2_PO_4_, and 72 mM K_2_HPO_4_; pH 7.0) containing antibiotics were inoculated with precultures and incubated at 37 °C and 150 rpm until they reached an optical density (OD_600_) of 0.6–0.8. Then, the expression of Gal-3 was induced by isopropyl 1-thio-β-d-galactopyranoside (0.5 mM). After 24 h, the cells were centrifuged (5000 rpm, 20 min, 4 °C) and frozen at −20 °C. For the purification of Gal-3, *E. coli* cells were suspended in an ice-cold equilibration buffer (20 mM Na_2_HPO_4_, 500 mM NaCl, and 20-mM imidazole; pH 7.4) and sonicated on ice (six 30 s cycles, 52% amplitude). After removing the cell debris (centrifugation at 13,400 rpm, 15 min, 10 °C), the supernatant was filtered through a 0.8 µm syringe filter and loaded on a HisTrap™ HP 5-mL column (GE Healthcare, Chicago, IL, USA), according to the manufacturer’s instructions, in an equilibration buffer. Gal-3 was eluted with elution buffer (20 mM Na_2_HPO_4_, 500 mM NaCl, and 500 mM imidazole; pH 7.4). The combined fractions containing galectin were dialyzed in SnakeSkin™ Dialysis Tubing (10 kDa MWCO, Thermo Fisher Scientific, Waltham, MA, USA) overnight against EPBS buffer (50 mM NaH_2_PO_4_, 150 mM NaCl, and 2 mM ethylenediaminetetraacetic acid; pH 7.5) and for an additional 4 h against PBS buffer (50 mM NaH_2_PO_4_ and 150 mM NaCl; pH 7.5). The usual yield was ca 7-10 g of cells per 1 L of medium and 5 mg of pure Gal-3 per 1 g of cells. After sterile filtration, Gal-3 (100–200 μM solutions) was stable for 1 to 2 months at 4 °C.

### 4.2. Preparation and Characterization of LacdiNAc Ligand for Gal-3

The disaccharide epitope LacdiNAc, which acts as a selective ligand for Gal-3, was prepared and structurally characterized as described in our recent work [25].

### 4.3. Cell Models

Human adipose tissue-derived stem cells (ADSCs) were isolated from a lipoaspirate obtained by liposuction from the thigh region of a patient (woman, aged 46 years) at a negative pressure of −200 mmHg. The isolation was conducted in compliance with the tenets of the Declaration of Helsinki for experiments involving human tissues and under the ethical approval issued by the Ethics Committee in Na Bulovce Hospital (Budínova 2, CZ 180 00 Prague 8). Written informed consent was obtained from the patient before the liposuction procedure. The ADSCs were isolated by a method described by Estes et al. (2010) [64] with the slight modifications reported in our earlier studies [65,66]. Briefly, the lipoaspirate was rinsed several times in phosphate-buffered saline (PBS) to remove the blood cells, digested by 0.1% type I collagenase in PBS for 1 h at 37 °C, and centrifuged. The obtained vascular–stromal fraction was washed two times, and it was filtered using a filter with 100-μm pores (Cell Strainer; BD Falcon, Franklin Lakes, NJ, USA). After the final washing and centrifugation, the cells were seeded into tissue culture polystyrene flasks (75 cm^2^; TPP, Trasadingen, Switzerland) at a density of 0.16 mL of the original lipoaspirate per cm^2^. The cells were expanded in Dulbecco’s modified Eagle’s medium (DMEM; Gibco, Thermo Fisher Scientific, Waltham, MA, USA) supplemented with 10% fetal bovine serum (FBS; Gibco, Thermo Fisher Scientific, Waltham, MA, USA), 40 µg/mL of gentamicin, and 10 ng/mL of FGF-2 (GenScript Biotech Co., Piscataway, NJ, USA, Cat. No. Z03116-1). In passage 2, the cells were characterized by flow cytometry (Accuri C6 Flow Cytometer, BD Biosciences, San Jose, CA, USA), which revealed the presence of standard surface markers of ASCs, namely CD105 (99.9%), CD90 (99.5%), CD73 (100%), and also, CD29 (100%). However, the ASCs were negative or almost negative for CD31 (0.5%), for CD34 (0.2%), CD45 (3.8%), and for CD146 (4.7%).

Human umbilical vein endothelial cells (HUVECs) were purchased from Lonza, Basel, Switzerland (Cat. No. C2517A, passage 3). The cells were expanded in endothelial cell growth medium 2 (EGM-2), which was prepared from endothelial cell basal medium 2 (EBM-2, PromoCell, Heidelberg, Germany, Cat. No. C-22111), supplemented with 1% antibiotic antimycotic solution (*v*/*v*, A5955, Sigma-Aldrich, St. Louis, MO, USA) and the growth medium 2 supplement pack (PromoCell, Heidelberg, Germany, Cat. No. C-39211) containing hydrocortisone, heparin, ascorbic acid, EGF, VEGF, IGF-1, FGF-2, and 2% FBS.

### 4.4. Natural Presence of Gal-3 in ADSCs and HUVECs

The natural presence of Gal-3 in the cell types used in this study was determined by immunofluorescence staining. The cells were fixed with 4% paraformaldehyde in phosphate-buffered saline (PBS) for 20 min at room temperature (RT). For staining of Gal-3 on the cell plasma membrane, the cells were incubated with PBS containing 1% *w/v* BSA for 20 min at RT. BSA was used for blocking the nonspecific-binding sites for the antibodies. After rinsing with PBS, the cells were incubated with a primary anti-Gal-3 antibody, produced in rabbit (Sigma-Aldrich, St. Louis, MO, USA; Cat. No. SAB4501746). The antibody was diluted in PBS at a ratio of 1:400, and the cells were incubated overnight at 4 °C. After washing with PBS, the cells were incubated for 1 h at RT in the dark with the secondary antibody Alexa Fluor^®^ 488 F(ab’)2 fragment of goat anti-rabbit IgG (H+L), Invitrogen, Carlsbad, CA, USA, Cat. No. A11070; diluted in PBS at a ratio of 1:400. To stain the intracellular Gal-3, the cells were permeabilized by incubation with PBS containing 1% *w/v* BSA and 0.1% Triton X-100 for 20 min at RT, followed by incubation with PBS containing 1% Tween for another 20 min at RT. Then, the cells were incubated with an anti-Gal-3 primary antibody (Sigma-Aldrich, St. Louis, MO, USA; Cat. No. SAB4501746) and Alexa Fluor^®^ 546 goat anti-rabbit IgG (H + L), Invitrogen, Carlsbad, CA, USA, Cat. No. A11010, to visualize the intracellular Gal-3. The presence and distribution of Gal-3 in the cells were then evaluated using the Andor Dragonfly 503 scanning disc confocal microscope equipped with a Zyla 4.2 PLUS sCMOS camera and objective HC PL APO 40×/1.10 W CORR CS2 (Andor Technology Ltd., Belfast, UK).

The cytosolic fraction of the ADSCs and HUVECs was isolated using the Qproteome cell compartment kit (Qiagen, Germantown, MD, USA) according to the manufacturer’s instructions. Briefly, after harvesting, approximately 4 × 10^6^ cells were washed twice with PBS, resuspended in 1 mL of ice-cold Lysis buffer, and swayed for 10 min at 4 °C. After centrifugation at 1000× *g* for 10 min at 4 °C, the supernatant (cytosolic proteins) was transferred into a fresh tube. The proteins were precipitated in acetone for 30 min on ice and dissolved in 200 μL of SDS-PAGE loading buffer. An equal amount of dissolved cytosolic proteins (18 μL of ADSCs and 20 μL of HUVECs) was loaded on 12% SDS-polyacrylamide gel and separated by electrophoresis (15 mA for a gel). Next, the proteins were transferred onto a 0.45-μm nitrocellulose membrane (100 V, 2 h, 4 °C). After washing with Tris-buffered saline with Tween 20 (TBS-T; 20 mM Tris, 14-mM NaCl, and 0.1% Tween 20; pH 8.0), the membrane was blocked with 10% milk in TBS-T for 2 h at room temperature. Subsequently, the membrane was incubated with a primary antibody against human Gal-3 (D4I2R, 1:1000, Cell Signaling Technology, Danvers, MA, USA) at 4 °C overnight in 5% milk in TBS-T and, then, with the HRP-conjugated anti-rabbit secondary antibody (1:5000, Abcam, Cambridge, UK). The signal was developed in the SuperSignal West Femto Maximum Sensitivity Substrate (Thermo Fisher Scientific, Waltham, MA, USA) and documented using the G:BOX Chemi XRQ gel doc system with GeneTools software (Syngene, Synoptics Group, Cambridge, UK).

### 4.5. Association of Free Gal-3 with the Cell Surface

HUVECs or ADSCs were grown to confluence in 96-well tissue culture polystyrene plates (TPP; Trasadingen, Switzerland, Cat. No. 92096). Then, the medium was removed, and the cells were incubated in 50 μL of fresh EGM-2 containing Gal-3 in concentrations of 0, 0.03, 0.1, 0.3, 1, 3, 10, or 30 μM for 1 h at 37 °C. The cells were then fixed with paraformaldehyde without permeabilization and were stained by immunofluorescence for Gal-3 bound on the cell cytoplasmic membrane, as described in Section 4.4, using the anti-Gal-3 antibody (EP2775Y) produced in rabbits (Abcam, Cambridge, UK; Cat. No. ab76245). The amount of Gal-3 on the cell surface was then estimated spectrophotometrically using a Synergy™ HT Multi-Mode Microplate reader (BioTek, Winooski, VT, USA) by measuring the fluorescence intensity of the stained Gal-3 bound to the cell membrane (Ex./Em. = 485/528 nm). The microphotographs were taken using an Olympus IX 71 epifluorescence microscope equipped with a DP 70 digital camera (Olympus, Tokyo, Japan).

Potential blocking of the association of Gal-3 with the cell membrane with LacdiNAc was then investigated. First, Gal-3 was diluted in the EGM-2 medium to the concentration of 30 μM (which proved to be the optimum concentration for the association of Gal-3 with the cell surface, as indicated by Figure 2 and Figure 3). Gal-3 was then incubated with LacdiNAc added to the same medium in 0, 0.01, 0.1, 1, 10, 50, or 100 mM concentrations for 1 h at 37 °C. After that, 50 μL of the media, with a combination of Gal-3 and each concentration of LacdiNAc, were added to the confluent layers of HUVECs in 96-well plates and were incubated with the cells for another 1 h at 37 °C.

The optimum concentration of LacdiNAc for blocking the association of Gal-3 with cells was then selected (1 mM; Figure 4) and used for further blocking experiments, in which Gal-3 and LacdiNAc were added to the cells in a different order. In these experiments, the cells were first incubated with 30 µM Gal-3 for 1 h, then were rinsed with PBS and incubated for an additional 1 h with 1 mM LacdiNAc or contrariwise. A group of cell samples was also incubated with a mixture of 30 µM Gal-3 and 1 mM LacdiNAc. Reference cell samples were incubated for 1 h either with Gal-3 (30 µM) alone or with LacdiNAc (1 mM) alone. The cells were then fixed with paraformaldehyde and stained for the cytoplasmic membrane-bound Gal-3. The amount of Gal-3 was then estimated spectrophotometrically, as mentioned above.

### 4.6. Cell Adhesion to Gal-3 Immobilized on Cultivation Substrate

For studies on the effect of Gal-3 as a cell adhesion ligand, this protein was adsorbed from a PBS solution overnight at 4 °C on the bottom of the wells in xCELLigence 96-well E-plates (E-plate view 96 PET, Cat. No. 300600910, ACEA Biosciences, San Diego, CA, USA). The PBS solutions contained 0, 0.1, 0.33, 1, 3.3, 10, or 33 μM Gal-3. For each concentration, six wells were used. One-half of these wells were blocked with 0.5% *w/v* bovine serum albumin (BSA) in PBS for 1 h at 37 °C, and the other half was left unblocked. The wells were then rinsed twice with PBS to remove unbound Gal-3 and BSA molecules and seeded with ADSCs or HUVECs at a density of 10^4^ cells/well in 200 μL of the cell culture medium. Pure DMEM and pure EBM-2 without any additives were used for the ADSCs and HUVECs, respectively. The cells in the plates were then incubated for 4 h at 37 °C in a humidified atmosphere containing 5% CO_2_ in the air, and the cell adhesion was monitored every 3 min for 4 h with a sensory xCELLigence system (Agilent Technologies, Waltham, MA, USA). This system enables the label-free, real-time monitoring of cell adhesion and growth based on electrical impedance, which is generated by the cells adhering to the bottoms of the sensory E-plates with interdigitated gold electrodes. During the first day after cell seeding, this impedance is generated mainly by cells initially attached to the well bottom and by the subsequent cell spreading, whereas, in the following days, the impedance is mainly attributed to the increase in the cell number, i.e., to the cell proliferation. The relative changes of the impedance in time are calculated by RTCA software (ACEA Biosciences, San Diego, CA, USA) as the cell index (CI). The cell index is defined as follows: CI = (impedance at time point n)—(impedance in the absence of cells)/(nominal impedance value). The normalized cell index value represents the cell index value of a sample normalized to the control sample (ctrl); thus, the cell index values of the samples are calculated proportionally to the cell index value of the control, which is set to 1.

The potential blocking of cell adhesion on substrate-immobilized Gal-3 with LacdiNAc was also investigated. The wells in an xCELLigence E-plate were preadsorbed with Gal-3 at a 1 μM concentration, which proved as the optimum concentration for the adhesion of both ADSCs and HUVECs (see below in Figure 5 and Table S1 in the Supplementary Materials). The wells were then blocked with 0.5% *w/v* BSA in PBS and were preincubated with 100 μL of pure DMEM containing LacdiNAc in concentrations of 0, 1, 2, 4, 10, 20, and 40 mM at 37 °C. After 1 h, the wells were seeded with ADSCs at a density of 10^4^ cells/well in 100 μL of pure DMEM containing the respective concentration of LacdiNAc. The cells were incubated at 37 °C in a humidified air atmosphere containing 5% CO_2_ in the xCELLigence system, where the cell adhesion was monitored every 15 min for 4 h.

### 4.7. The Role of Integrin Adhesion Receptors in Gal-3-Mediated Cell Adhesion

To study the role of integrin receptors in Gal-3-mediated cell adhesion, the wells in an xCELLigence E-plate were coated with 1 μM Gal-3 and blocked with 0.5% *w/v* BSA as described above. The ADSCs or HUVECs were trypsinized and resuspended in a pure medium to a final concentration of 10^5^ cells/mL. The cell suspension was then incubated in wells of non-tissue culture-treated 24-well polystyrene plates (Corning Inc., Corning, NY, USA, Cat. No. 351147) at 37 °C for 30 min in a rotary shaker (300 rpm) with ethylenediaminetetraacetic acid (EDTA), a potent chelator of calcium, which is indispensable for the adhesion function of integrin receptors. The cells were incubated with EDTA in 0.1, 1, and 10 mM concentrations. To observe the role of the RGD-binding integrins in Gal-3-mediated cell adhesion, the cells were incubated with the GRGDSP peptide (SCP0157, Sigma-Aldrich, St. Louis, MO, USA) at the concentration of 200 µM. The GRADSP peptide (SCP0156, Sigma-Aldrich, St. Louis, MO, USA) was used as a negative control. To specify the type of integrin receptor that was involved in Gal-3-mediated cell adhesion, the cells were incubated with antibodies against the integrin subunit αV (Millipore, Burlington, MA, USA, MAB2021Z, clone AV1, which binds to the αV integrin subunit [67] and blocked the adhesion to various substrates [68]); subunit α3 (Millipore, Burlington, MA, USA, MAB1952Z, clone P1B5, which specifically binds to the α3 subunit and inhibits the adhesion to collagen, fibronectin, or laminin [69]); subunit β1 (Millipore, Burlington, MA, USA, MAB2253, clone 6S6, which binds specifically to the β1 integrin subunit [70] and blocks the adhesion to collagen or fibronectin [71]); subunit β3 (Millipore, Burlington, MA, USA, MAB2023Z, clone B3A, which is specific to the β3 subunit and inhibits cell adhesion [72,73]); against integrin αVβ3 (Millipore, Burlington, MA, USA, MAB1976, clone LM609, which specifically recognizes an epitope on the αVβ3 heterodimer and prevents the adhesion to vitronectin, fibrinogen, or the von Wilebrand factor [74]); integrin α5β1 (Millipore, Burlington, MA, USA, MAB1969, clone JBS5, which recognizes an epitope on the α5β1 integrin heterodimer [75] and inhibits the adhesion to fibronectin [76]); or integrin α2β1 (Millipore, Burlington, MA, USA, MAB1998, clone BHA2.1, which specifically recognizes the α2β1 heterodimer, is unable to bind the integrin receptor-lacking I-domain, and blocks the adhesion to collagen or laminin [77]); or with an αVβ1 integrin inhibitor (MedChemExpress, Monmouth Junction, NJ, USA, HY-100445A). Mouse monoclonal IgG1 (Millipore, Burlington, MA, USA, MABC002) was used as the isotype control. The antibody against the αV integrin subunit was used in the 1:25 dilution, and the other antibodies were used in a concentration of 20 μg/mL. The αVβ1 integrin inhibitor was used in the concentrations of 0.01, 0.1, and 10 μg/mL. The cells were then seeded in wells of xCELLigence E-plates into 100 μL of medium (10^4^ cells/well), and the initial cell adhesion was measured for 4 h.

To analyze the expression of integrin adhesion receptors on the cell surface, the antibodies against integrin subunits αV (MAB2021Z), α3 (MAB1952Z), β1 (MAB2253), β3 (MAB2023Z), and against integrins αVβ3 (MAB1976), α5β1 (MAB1969), and α2β1 (MAB1998), were used. All these antibodies were purchased from Millipore, Burlington, MA, USA. The ADSCs and HUVECs were trypsinized and resuspended in PBS containing 0.5% *w/v* BSA. The cells were incubated with primary antibodies on ice for 20 min. The antibody against the αV integrin subunit was used in the dilution 1:50, and the other primary antibodies were used in a concentration of 10 μg/mL. Then, the cells were centrifuged and washed in 0.5% *w/v* BSA and incubated with anti-mouse IgG conjugated with Alexa Fluor 488 (10 μg/mL, Cat. No. A11017, Thermo Fisher Scientific, Waltham, MA, USA) for another 15 min on ice. The cells were centrifuged and washed in 0.5% *w/v* BSA. The presence of integrins on the cell surface was analyzed using a NovoCyte^®^ Flow Cytometer (ACEA Biosciences, Agilent, Santa Clara, CA, USA). At least 30,000 ADSCs and 15,000 HUVECs were analyzed per sample. To determine the background fluorescence signal, the cells were left unstained or incubated with nonspecific mouse monoclonal IgG1 (Millipore, Burlington, MA, USA, MABC002) and, then, with the secondary antibody.

### 4.8. Comparison of Gal-3 with Other Adhesion Ligands and Cell Morphology

To compare the cell adhesion-promoting ability of Gal-3 with the other extracellular proteins commonly used for material surface coatings, 96-well glass plates (Cellvis, Mountain View, CA, USA, P96-1.5H-N) were preadsorbed with Gal-3 (1 μM in PBS), fibronectin (20 μg/mL in PBS), or collagen I (50 µg/mL in 0.02 M acetic acid) overnight at 4 °C and then blocked with 0.5% *w/v* BSA for 1 h at 37 °C. The ADSCs or HUVECs were seeded at a density of 7.5 × 10^3^ cells/well in 200 μL of the pure medium (DMEM or EBM-2, respectively). After 4 h of incubation at 37 °C, the cells were fixed, permeabilized, and stained to visualize vinculin-containing focal adhesions in the cell membrane and filamentous actin (F-actin) cytoskeleton.

For visualizing the vinculin-containing focal adhesions, the cells were fixed and permeabilized as described in Section 2.4. The cells were then stained with an antibody against vinculin, an integrin-associated focal adhesion protein (mouse monoclonal antibody, Cat. No. V9131, Sigma-Aldrich; dilution 1:400) overnight at 4 °C. The cells were washed with PBS and were incubated with anti-mouse IgG conjugated with Alexa Fluor 488 (Cat. No. A11017, Thermo Fisher Scientific, Burlington, MA, USA; dilution 1:400) for 1 h at RT in the dark. At the same time, the F-actin cytoskeleton was stained with phalloidin-TRITC (0.1 μg/mL in PBS, Sigma-Aldrich, St. Louis, MO, USA), and the cell nuclei were counterstained with Hoechst 33,258 (10 μg/mL in PBS) for 1 h at RT (both dyes were added into the secondary antibody solution).

The stained cells were observed through the Olympus epifluorescence microscope IX71 (DP71 digital camera, objective magnification 20×, 40×, or 100×). The morphology characteristics, such as cell area, perimeter, circularity, and solidity (i.e., a parameter inversely correlated with the presence of protrusions on the cell surface), were evaluated from microphotographs using ImageJ software (National Institutes of Health, Bethesda, MD, USA; version FIJI 2.0.0-rc-68/1.52 h).

### 4.9. Statistics

The data in the graphs are shown as the mean ± SD from three wells. The data depicted in Figure S9 in the Supplementary Materials (comparisons of the morphology characteristics of the cells) were statistically analyzed using ANOVA on Ranks, Dunnʹs method, *p* ≤ 0.05. The data in the remaining figures were analyzed with the use of one-way ANOVA, Holm-Sidak test, *p* ≤ 0.05. The data were analyzed with SigmaPlot 14.0 software (Systat Software Inc., San José, CA, USA).

## 5. Conclusions

We found that Gal-3 is naturally present on the surface of human adipose tissue-derived stem cells (ADSCs) and human umbilical vein endothelial cells (HUVECs). Gal-3 from extracellular environments, e.g., from the culture medium, can associate with these cells in a concentration-dependent manner, and this association can be significantly reduced by LacdiNAc, a selective ligand for Gal-3, bound to the canonical binding site on the carbohydrate recognition domain (CRD) of the Gal-3 molecule. Moreover, LacdiNAc can detach the newly associated Gal-3 from the cells in a concentration-dependent manner. These results indicate that free extracellular Gal-3 can associate with cells through its CRD domain.

Another important finding of this study is that Gal-3 acts as an extracellular ligand mediating cell adhesion. After the adsorption on tissue culture well plates and blocking the nonspecific binding sites with BSA (which is a nonadhesive for cells), Gal-3 promoted the initial adhesion of ADSCs and HUVECs, and LacdiNAc was not able to block this adhesion when incubated with the adsorbed Gal-3 before cell seeding or when added into the cell culture medium. Therefore, cell adhesion to extracellular Gal-3 seems to be mediated by a binding site different from the carbohydrate-binding site on the CRD, which has not been observed so far and should be further investigated. On the cell membrane, the cell adhesion to the substrate-bound Gal-3 is mediated, at least partially, by integrins with the αV and β1 chains—namely, with the α5β1, αVβ3, and αVβ1 integrins, as revealed by integrin-blocking experiments with specific anti-integrin antibodies.

## Data Availability

The data presented in this study are available on request from the corresponding authors.

## Supplementary Materials

The following are available online at https://www.mdpi.com/article/10.3390/ijms22105144/s1: Figure S1: Spreading of ADSCs and HUVECs on wells of E-plates in the xCELLigence system four hours after seeding. Figure S2: The effect of LacdiNAc on the spreading of ADSCs four hours after seeding into untreated wells or wells preadsorbed with 1 μM Gal-3 and blocked with BSA. Figure S3: Role of integrins in the spreading of ADSCs and HUVECs on substrate-immobilized Gal-3 (four hours after seeding). Figure S4: Cell surface expression of integrins on ADSCs. Figure S5: Cell surface expression of integrins on HUVECs. Figure S6: Adhesion of ADSCs on wells one hour after seeding in a medium containing various concentrations of the αVβ1 inhibitor. Figure S7: Fluorescence staining of ADSCs and HUVECs adhered to various protein-coated glass surfaces 4 h after cell seeding (objective magnification 40×). Figure S8: Fluorescence staining of ADSCs and HUVECs adhered to various protein-coated glass surfaces four hours after cell seeding (objective magnification 100×). Figure S9: The morphology characteristics of ADSCs and HUVECs adhered to various protein-coated glass surfaces four hours after seeding.

## Abbreviations

ADSC	Adipose tissue-derived stem cells
BSA	Bovine serum albumin
CI	Cell index
CRD	Carbohydrate recognition domain
EBM-2	Endothelial cell basal medium-2
ECM	Extracellular matrix
EGF	Epidermal growth factor
EGM-2	Endothelial cell growth medium-2
EPC	Endothelial progenitor cell
FBS	Fetal bovine serum
FGF-2	Fibroblast growth factor-2
Gal-3	Galectin-3
GRGDSP	Gly-Arg-Gly-Asp-Ser-Pro integrin-blocking RGD-based peptide
HIV	Human immunodeficiency virus
HPMA	*N*-(2-Hydroxypropyl) methacrylamide
HUVEC	Human umbilical vein endothelial cell
IGF-1	Insulin-like growth factor-1
LacdiNAc	2-Acetamido-2-deoxy-β-d-galactopyranosyl-(1→4)-2-acetamido-2-deoxy-d-glucopyranose
LacNAc	β-d-Galactopyranosyl-(1→4)-2-acetamido-2-deoxy-d-glucopyranose
PBS	Phosphate-buffered saline
RGD	Arg-Gly-Asp binding motif
RT	Room temperature
