# Supplementary material for: Interaction between Galectin-3 and Integrins Mediates Cell-Matrix Adhesion in Endothelial Cells and Mesenchymal Stem Cells
Source: Int J Mol Sci. 2021 May 13;22(10):5144. doi: 10.3390/ijms22105144 (PMC8152275; doi:10.3390/ijms22105144)
Supplement: Supplementary file 1 [file ijms-22-05144-s001.zip › ijms-1139774-supplementary.pdf]

International Journal of *Molecular Sciences*

MDPI

*Supplementary Materials*

# Interaction between galectin-3 and integrins mediates cell-matrix adhesion in endothelial cells and mesenchymal stem cells

**Antonín Sedlář[1,2], Martina Trávníčková[1], Pavla Bojarová[3,4*], Miluše Vlachová[3], Kristýna Slámová[3], Vladimír Křen[3], Lucie Bačáková[1*]**

[1] Laboratory of Biomaterials and Tissue Engineering, Institute of Physiology of the Czech Academy of Sciences, Vídeňská 1083, CZ 142 20 Prague 4, Czech Republic; antonin.sedlar@fgu.cas.cz (A.S.), martina.travnickova@fgu.cas.cz (M.T.), lucie.bacakova@fgu.cas.cz (L.B.)

[2] Department of Physiology, Faculty of Science, Charles University, Viničná 7, CZ 128 44 Prague 2, Czech Republic

[3] Laboratory of Biotransformation, Institute of Microbiology of the Czech Academy of Sciences, Vídeňská 1083, CZ 142 20 Prague 4, Czech Republic; bojarova@biomed.cas.cz (P.B.), miluse.vlachova@biomed.cas.cz (M.V.), slamova@biomed.cas.cz (K.S.), kren@biomed.cas.cz (V.K.)

[4] Department of Health Care Disciplines and Population Protection, Faculty of Biomedical Engineering, Czech Technical University in Prague, Nám. Sítná, CZ 272 01 Kladno, Czech Republic

* Correspondence: bojarova@biomed.cas.cz; Tel.: +420 296442360 (P.B.); lucie.bacakova@fgu.cas.cz; Tel.: +420 296443743 (L.B.)

**Abstract:** Galectin-3 (Gal-3) is a β-galactoside-binding protein that influences various cell functions, including cell adhesion. We focused on the role of Gal-3 as an extracellular ligand mediating cell-matrix adhesion. We used human adipose tissue-derived stem cells and human umbilical vein endothelial cells that are promising for vascular tissue engineering. We found that these cells naturally contained Gal-3 on their surface and inside the cells. Moreover, they were able to associate with exogenous Gal-3 added to the culture medium. This association was reduced with a β-galactoside LacdiNAc (GalNAcβ1,4GlcNAc), a selective ligand for Gal-3, which binds to the carbohydrate recognition domain (CRD) in the Gal-3 molecule. This ligand was also able to detach Gal-3 newly associated with cells but not Gal-3 naturally present on cells. In addition, Gal-3 preadsorbed on plastic surfaces acted as an adhesion ligand for both cell types, and the cell adhesion was resistant to blocking with LacdiNAc. This result suggests that the adhesion was mediated by a binding site different from the CRD. Blocking of integrin adhesion receptors on cells with specific antibodies revealed that the cell adhesion to the preadsorbed Gal-3 was mediated, at least partially, by β1 and αV integrins, namely α5β1, αVβ3, and αVβ1 integrins.

**Keywords:** galectin; HUVEC; ADSC; integrin; carbohydrate

A

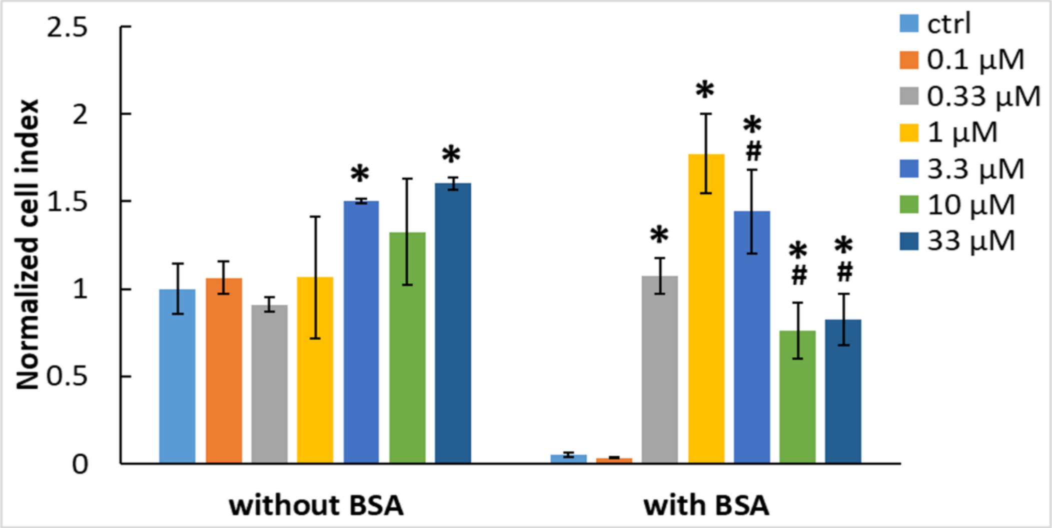


B

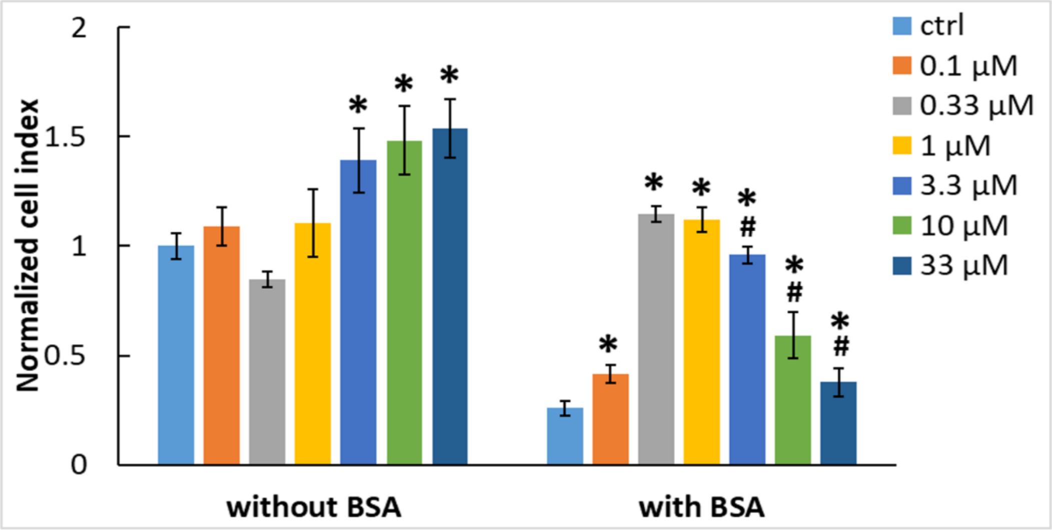


**Figure S1**. Spreading of ADSCs **(A)** and HUVECs **(B)** on wells of E-plates in xCELLIgence system 4 h after seeding. The wells were adsorbed with various concentrations of Gal-3 (0.1 – 33 µM) either without blocking or with blocking using 0.5 % w/v BSA. Cell index values were normalized to the control cell sample without adsorbed Gal-3 and BSA (ctrl without BSA). Mean ± SD (n = 3). Holm-Sidak test, $p \leq 0.05$. The cell samples were statistically compared either within the group without BSA or within the group with BSA. * - statistically significant difference in comparison with control samples without adsorbed Gal-3 (ctrl), # - statistically significant difference in comparison with the sample exhibiting the highest average value of cell index.

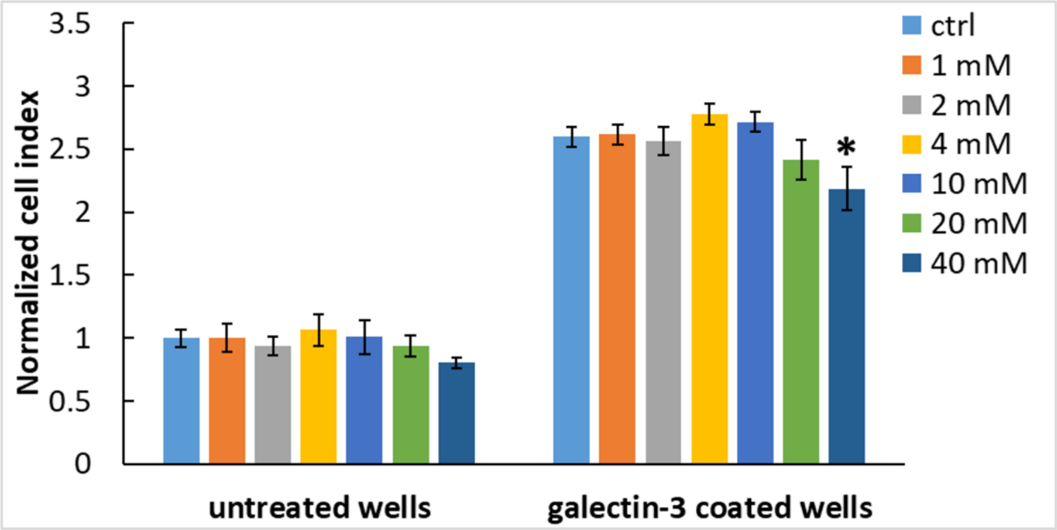


**Figure S2.** The effect of LacdiNAc on spreading of ADSCs 4 h after seeding into untreated wells or wells pre-adsorbed with 1 μM Gal-3 and blocked with BSA. Before cell seeding, both groups of wells were pre-incubated with LacdiNAc in concentrations from 1 to 40 mM. Wells without LacdiNAc served as control samples (ctrl). Cell index values were normalized to the sample without Gal-3 adsorption and without LacdiNAc in the medium (ctrl - untreated wells). Mean ± SD from 3 wells. Holm-Sidak test, $p \leq 0.05$. The cell samples were statistically compared either within the group of untreated wells or within the group of Gal-3-adsorbed wells. * - statistically significant difference in comparison with control cells (ctrl).

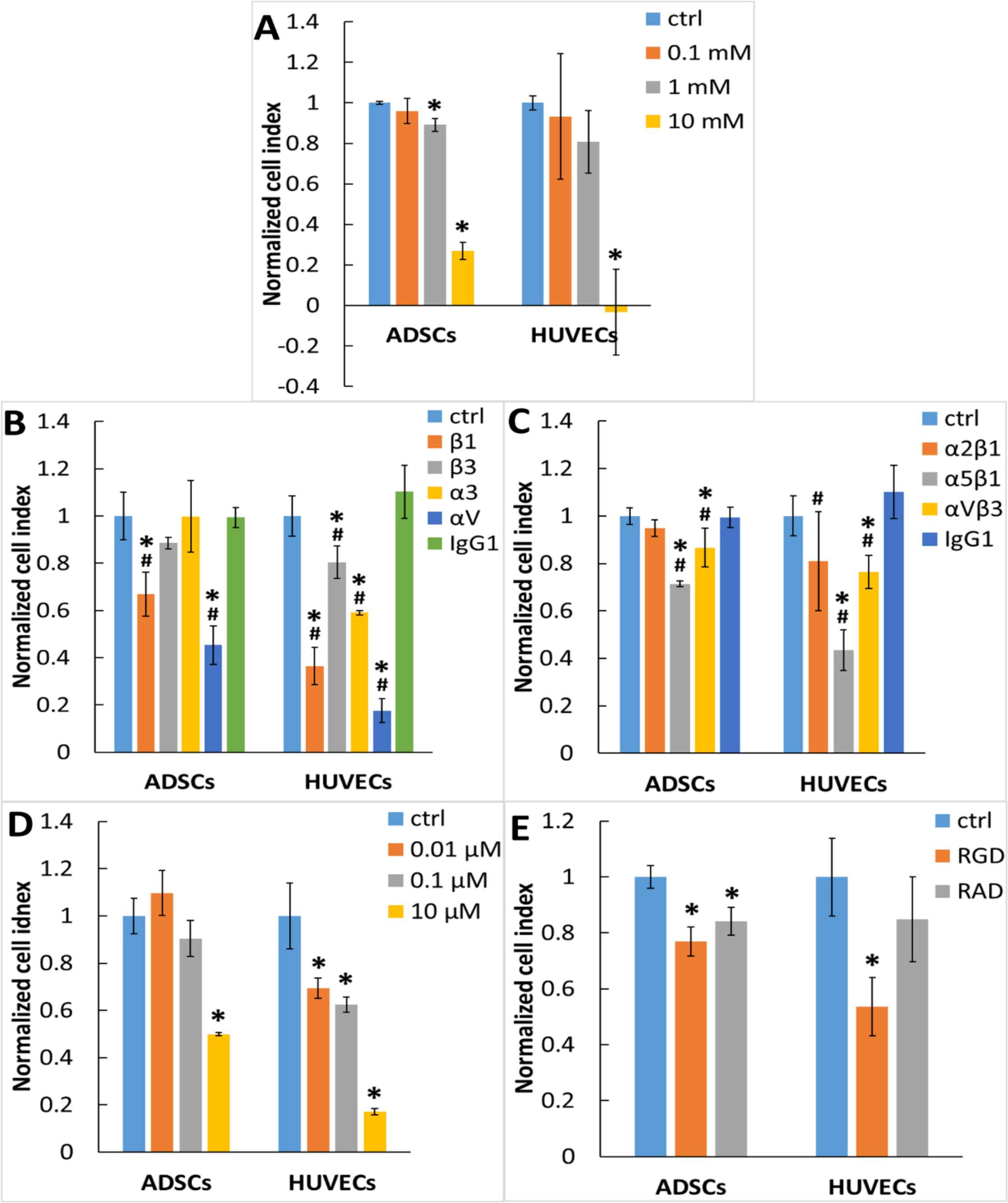


**Figure S3.** Role of integrins in the spreading of ADSCs and HUVECs on substrate-immobilized Gal-3 (4 h after seeding). **(A)** The cells were seeded in a medium containing EDTA in concentrations ranging from 0.1 to 10 mM. **(B)** The cells were seeded in a medium containing antibodies against β1, β3, α3 and αV integrin subunits. The antibody against αV integrin subunit was used in dilution 1:25, the other antibodies were used in a concentration of 20 μg/mL. Non-specific mouse IgG1 was used as an isotype control. **(C)** The cells were seeded in a medium containing antibodies against α2β1, α5β1, or αVβ3 integrin receptors. Non-specific mouse IgG1 was used as an isotype control. Antibodies were used in a concentration of 20 μg/mL. **(D)** The cells were seeded in a medium containing αVβ1 integrin inhibitor in concentrations ranging from 0.01 to 10 μM. **(E)** The cells were seeded in a medium containing GRGDSP peptide. GRADSP peptide was used as a negative control. Both peptides were used in a concentration of 200 μM. Prior to cell seeding, the wells were coated with 1 μM Gal-3 and blocked with 0.5 % w/v BSA. Cell index values were normalized to the untreated sample in a pure medium without any additive (ctrl). Mean ± SD from 3 wells. Holm-Sidak test, $p \leq 0.05$. The samples were statistically compared within the group of the indicated cell type. * - statistically significant difference in comparison with control cells (ctrl). # - statistically significant difference in comparison with cells incubated with isotype control (IgG1).

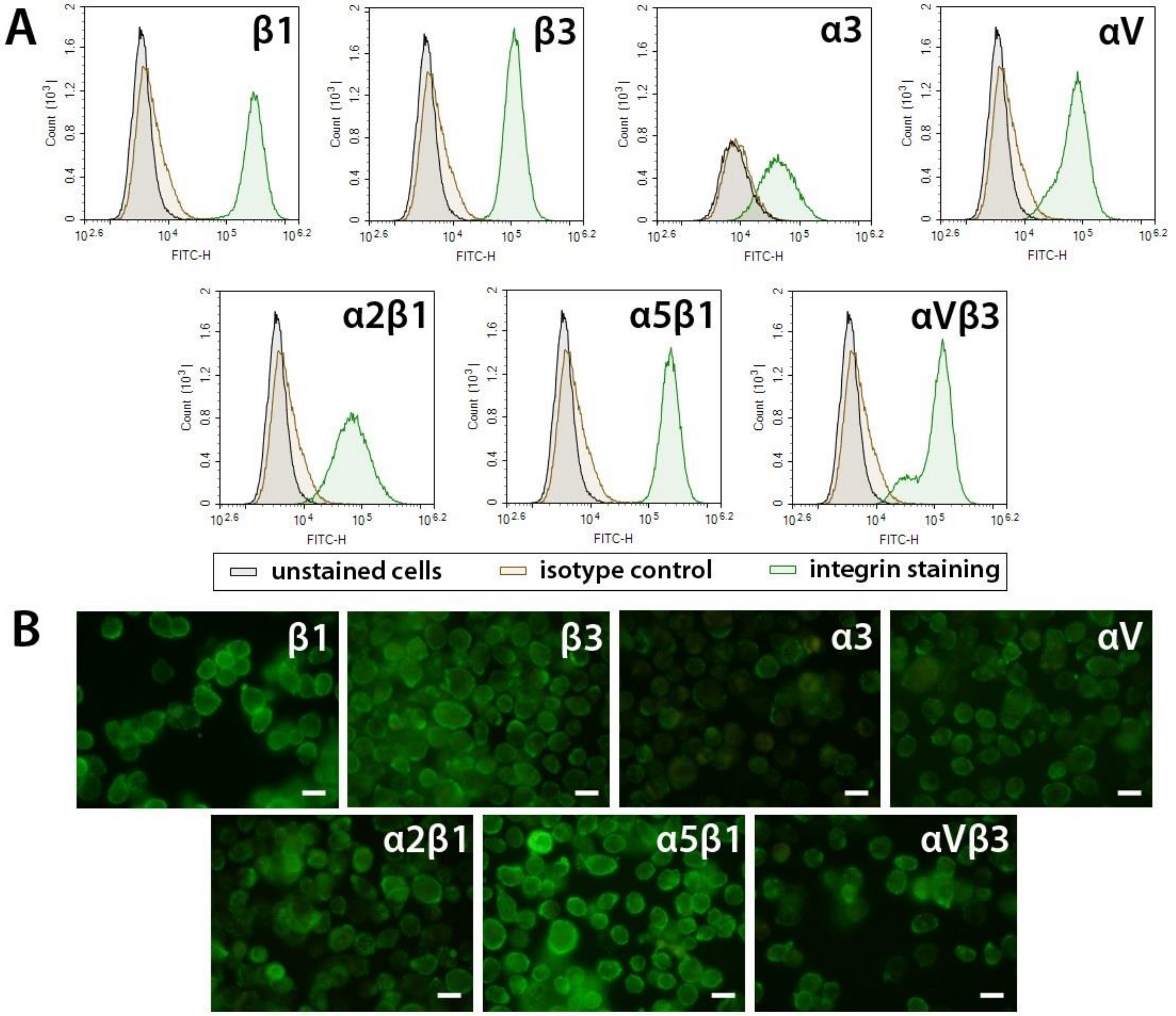


**Figure S4.** Cell surface expression of integrins on ADSCs. The presence of integrin subunits β1, β3, α3, αV, and of integrins α2β1, α5β1, αVβ3 was determined. **(A)** Flow cytometry analysis. Cells were incubated with antibodies against respective integrins (green histograms) or non-specific mouse IgG1 – isotype control (brown histograms), followed by incubation with anti-mouse IgG conjugated with Alexa Fluor 488. The fluorescence signal of unstained cells (black histograms) was determined as well. **(B)** Microphotographs of the cells in suspension incubated with the indicated anti-integrin antibodies, and with anti-mouse IgG conjugated with Alexa Fluor 488. Olympus IX 71 microscope, DP 70 digital camera, obj. 40×, scale bar 20 μm.

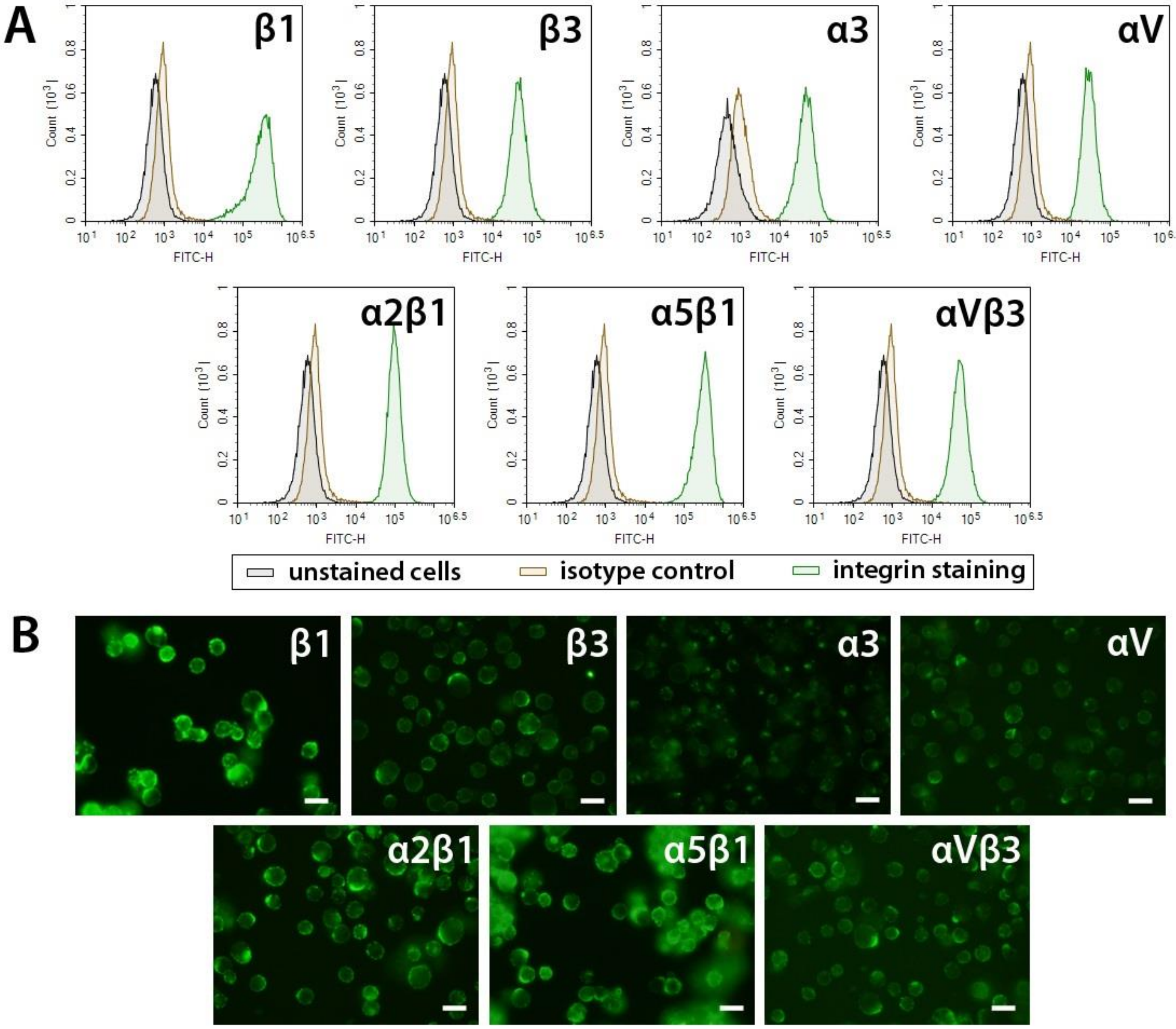


**Figure S5.** Cell surface expression of integrins on HUVECs. The presence of integrin subunits β1, β3, α3, αV, and of integrins α2β1, α5β1, αVβ3 was determined. **(A)** Flow cytometry analysis. The cells were incubated with antibodies against respective integrins (green histograms) or non-specific mouse IgG1 – isotype control (brown histograms), followed by incubation with anti-mouse IgG conjugated with Alexa Fluor 488. The fluorescence signal of unstained cells (black histograms) was determined as well. **(B)** Microphotographs of the cells in suspension incubated with the indicated anti-integrin antibodies, and with anti-mouse IgG conjugated with Alexa Fluor 488. Olympus IX 71 microscope, DP 70 digital camera, obj. 40×, scale bar 20 μm.

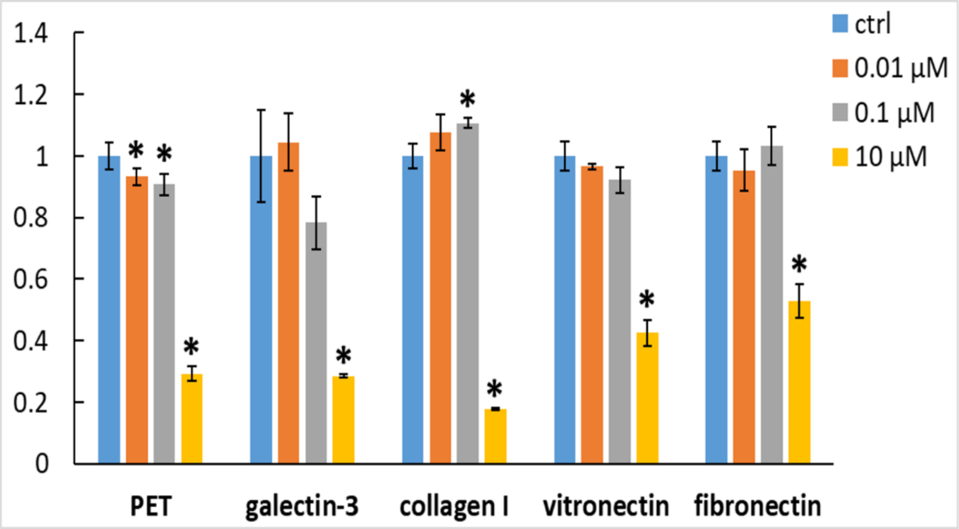


**Figure S6.** Adhesion of ADSCs on wells 1 h after seeding in a medium containing various concentrations of $\alpha V\beta 1$ inhibitor. The wells were pre-coated with galectin-3 (1 µM), collagen I (50 µg/mL), vitronectin (0.4 µg/mL) or fibronectin (20 µg/mL) and blocked with 0.5 % w/v BSA. As a control, some of the wells were left uncoated and non-blocked (PET). Cell index values were normalized to the samples containing no inhibitor (ctrl). Mean ± SD from 3 wells. Holm-Sidak test, $p \leq 0.05$. The cell samples were statistically compared within the groups indicated on the x-axis. * - statistically significant difference in comparison with control cells (ctrl).

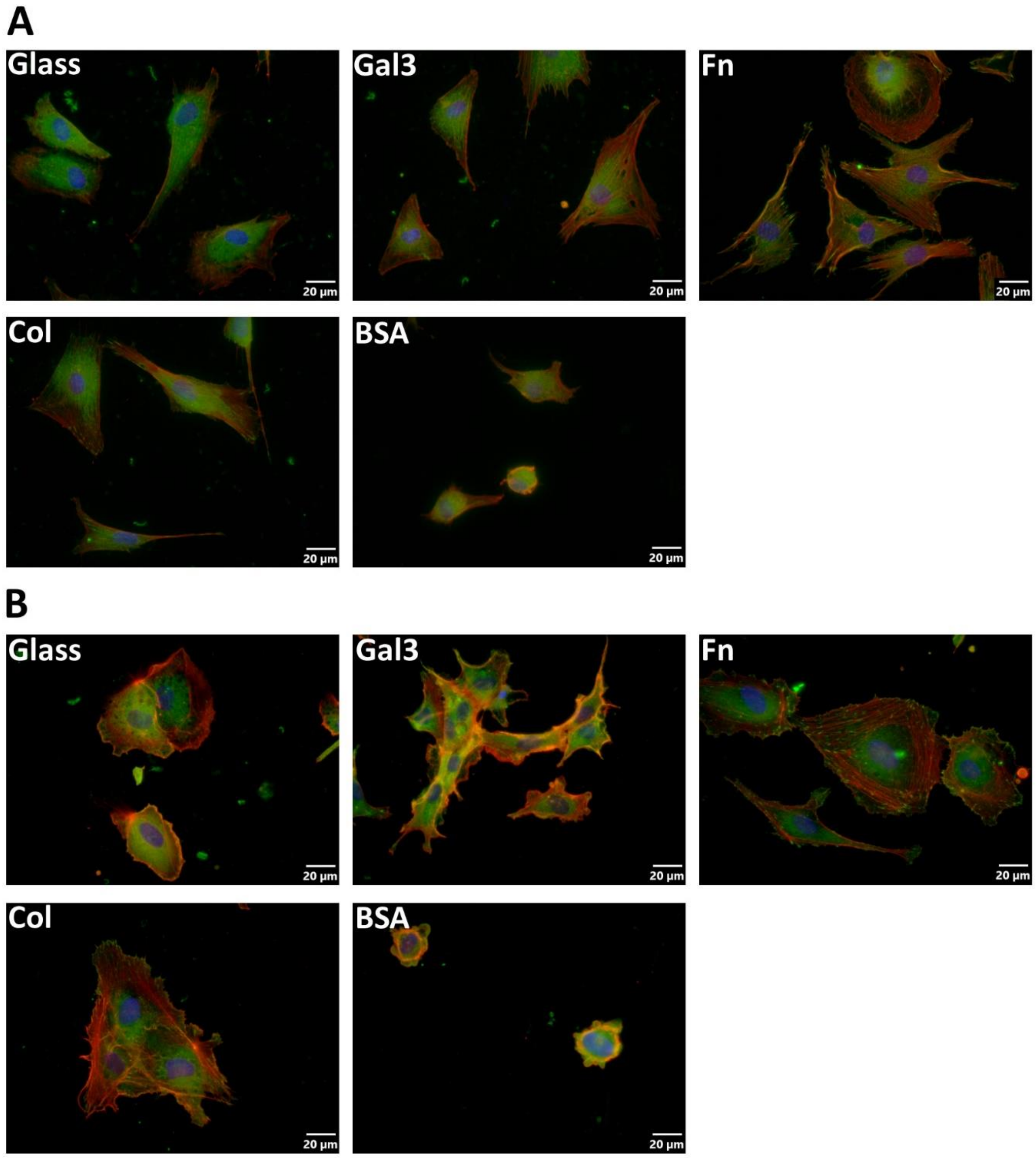


**Figure S7.** Fluorescence staining of ADSCs **(A)** and HUVECs **(B)** adhered to various protein-coated glass surfaces 4 h after seeding (objective magnification 40×). Wells on 96-well glass plate were left untreated (Glass), coated with Gal-3 (Gal3; 1 μM), fibronectin (Fn, 20 μg/mL) or collagen I (Col, 50 μg/mL) overnight at 4 °C and blocked with 0.5 % w/v BSA (1h; 37 °C), or coated only with 0.5 % w/v BSA (1h; 37 °C). Cells were stained with antibody against vinculin (green) and TRITC-conjugated phalloidin to visualize the F-actin cytoskeleton. Cell nuclei were counterstained with Hoechst 33258. Olympus IX 71 microscope, DP 70 digital camera, obj. 40×, scale bar 20 μm.

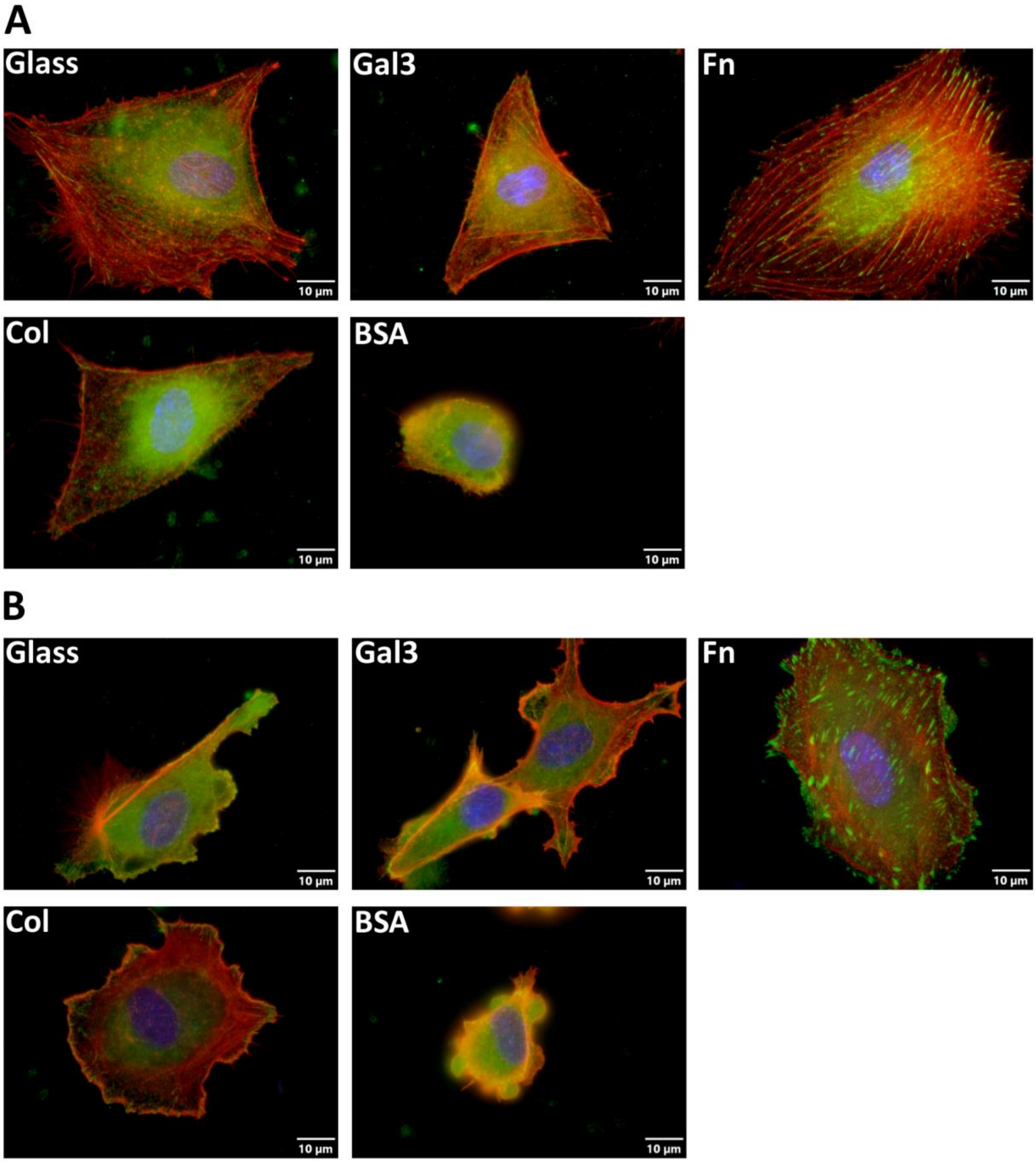


**Figure S8.** Fluorescence staining of ADSCs **(A)** and HUVECs **(B)** adhered to various protein-coated glass surfaces 4 h after seeding (objective magnification 100×). Wells on 96-well glass plate were left untreated (Glass), coated with Gal-3 (Gal3; 1 µM), fibronectin (Fn, 20 µg/mL) or collagen I (Col, 50 µg/mL) overnight at 4 °C and blocked with 0.5 % w/v BSA (1h; 37 °C), or coated only with 0.5 % w/v BSA (1h; 37 °C). Cells were stained with antibody against vinculin (green) and TRITC-conjugated phalloidin to visualize the F-actin cytoskeleton. Cell nuclei were counterstained with Hoechst 33258. Olympus IX 71 microscope, DP 70 digital camera, obj. 100×, scale bar 10 µm.

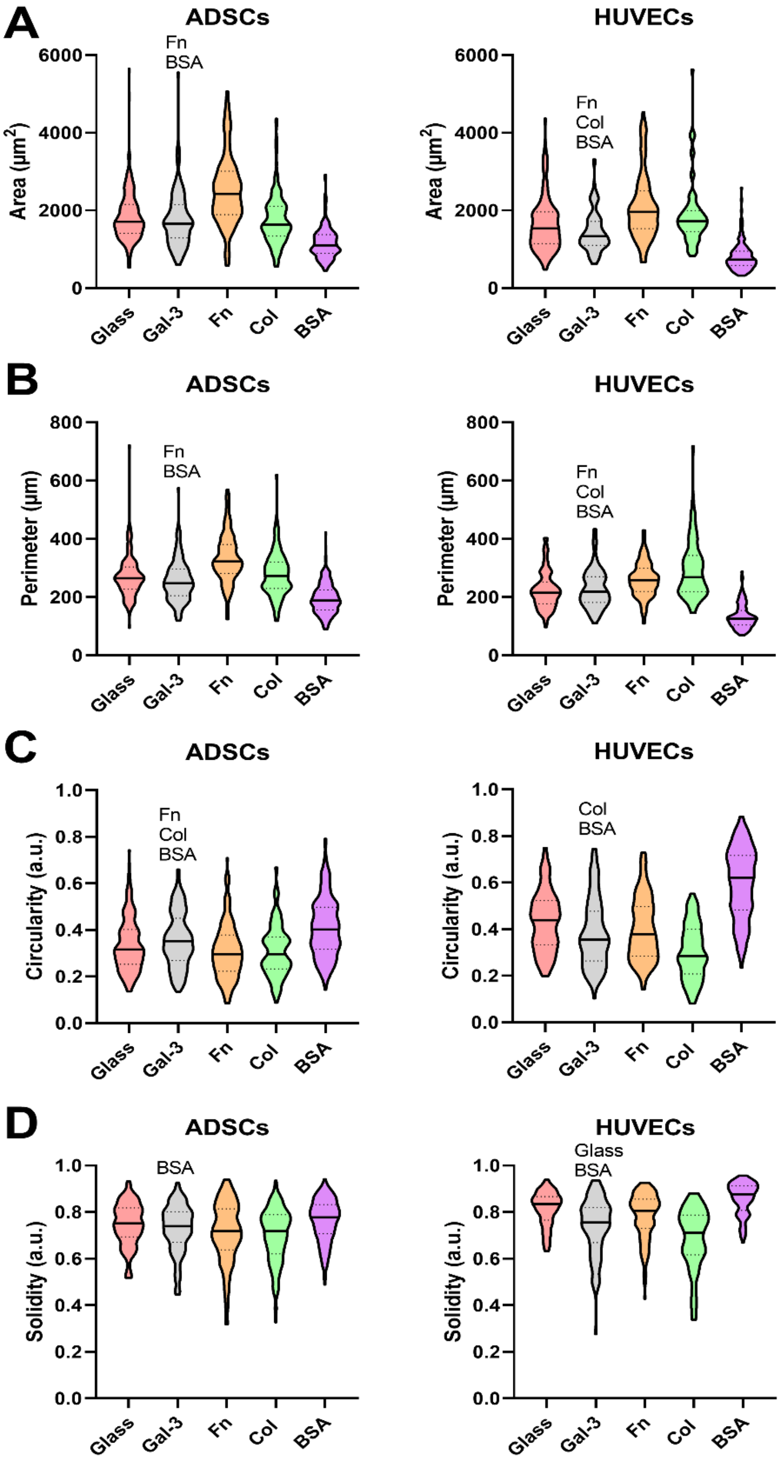


**Figure S9.** The morphology characteristics of ADSCs and HUVECs adhered to various protein-coated glass surfaces 4 h after seeding. Wells on 96-well glass plate were left untreated (Glass), coated with Gal-3 (Gal-3; 1 µM), fibronectin (Fn, 20 µg/mL) or collagen I (Col, 50 µg/mL) overnight at 4 °C and blocked with 0.5 % w/v BSA (1 h; 37 °C), or coated only with 0.5 % w/v BSA (1 h; 37 °C). Cells were stained with TRITC-conjugated phalloidin to visualize the F-actin cytoskeleton. Cell area **(A)**, perimeter **(B)**, circularity **(C)** and solidity **(D)** were evaluated from microphotographs using ImageJ software. The data are presented as violin plots with a solid line representing mean and with dotted lines representing the first and the third quartile. ANOVA on Ranks, Dunn's method, $p \leq 0.05$. Only the statistically significant differences between Gal-3 and other samples are depicted.
